# FKBP5-CCL5 interaction promotes neuroinflammation and neuronal apoptosis in ischemic stroke by regulating the MAPK pathway and enhancing NET formation

**DOI:** 10.3389/fimmu.2025.1609989

**Published:** 2025-09-30

**Authors:** Zhongchen Li, Tengkun Yin, Hongyang Guo, Zhenxing Liu, Peijian Wang, Chao Liu, Qingbo Wang, Meng Zhang, Yilei Xiao, Jiyue Wang, Jiheng Hao, Liyong Zhang

**Affiliations:** 1 Department of Neurosurgery, Liaocheng People’s Hospital, Liaocheng, Shandong, China; 2 Department of Neurosurgery, Qilu Hospital and Institute of Brain and Brain-Inspired Science, Cheeloo College of Medicine, Shandong University, Shandong, China

**Keywords:** ischemic stroke, FKBP5, CCL5, NET, MAPK

## Abstract

**Background:**

The pathophysiology of ischemic stroke is not fully elucidated. Upregulation of FKBP5 in brain ischemia/reperfusion injury has been found to be associated with the severity of ischemic and reperfusion damage. However, its specific role in ischemic stroke progression remains unclear.

**Methods:**

A total of 40 ischemic stroke patients and 40 age- and sex-matched healthy donors (HDs) were enrolled in this work to evaluate the expression of FKBP5, the formation of neutrophil extracellular trap (NET), and the correlation between NET and stroke. Moreover, transient middle cerebral artery occlusion (tMCAO) mouse model with 60 min occlusion (n = 15/group) was treated with CI-amidine to demonstrate the effect of NET on the stroke-related brain injury. Primary neurons were isolated from mouse brain tissue to evaluated the effect of NET on neuronal apoptosis through flow cytometry the TUNEL assays. In addition, BV2 microglial cells were transfected with FKBP5 overexpression and knockdown vectors. The microglial cells polarization, neutrophil NETs formation, and the underlying molecular action mechanism were measured. For specific methods: detected the levels of H3cit, MPO-DNA, IL-1β, IL-10, TNFα, and iNOS by ELISA; Pathological staining was viewed for the neuronal morphological changes; flow cytometry and TUNEL staining were viewed for the neuronal cell apoptosis; detected the protein levels of FKBP5, CD206, CCL5, and MAPK pathway by western blot.

**Results:**

In this study, we observed significant upregulation of FKBP5 in ischemic stroke patients, which was associated with increased expression of NET markers, such as H3cit and MPO-DNA complexes. This upregulation correlated with stroke severity and outcomes. In a transient middle cerebral artery occlusion (tMCAO) mouse model, treatment with the NET inhibitor CI-amidine significantly reduced brain injury, infarct size, and NET marker levels, suggesting therapeutic potential in targeting NETs. We further found that FKBP5 modulates microglial polarization towards a pro-inflammatory M1 phenotype and promotes NET formation. FKBP5 interacts with CCL5, enhancing MAPK pathway activation and increasing pro-inflammatory cytokine production, including TNF-α and IL-1β. Intervention with the MAPK pathway inhibitor AZD6244 effectively inhibited these effects.

**Conclusions:**

The current findings suggest that FKBP5 might modulate CCL5-mediate p38 MAPK signaling and NET formation, thereby contributing to post-stroke neuroinflammation and neuronal apoptosis. Further prospective research is needed to verify the potential of FKBP5 as therapeutic targets for ischemic stroke treatment.

## Introduction

Stroke is a common and severe condition that poses a significant threat to human health, characterized by high incidence, disability, and mortality rates (1). Recent studies have increasingly underscored the critical role of inflammatory responses in the pathophysiology of stroke, particularly ischemic stroke (2, 3). Understanding the underlying mechanisms of inflammation in ischemic stroke is crucial for developing effective therapeutic strategies.

Recent studies have highlighted a significant role of Neutrophil Extracellular Traps (NETs) in the pathophysiology of ischemic stroke (4, 5). NETs are web-like structures composed of DNA and various proteins that are released by activated neutrophils. During NET formation, neutrophils release their DNA along with granule proteins. Citrullination of histone H3 by the enzyme peptidyl arginine deiminase 4 (PAD4) is a crucial step in this process, as it is required for chromatin decondensation and DNA release (6). NET formation is considered neutrophil-specific and distinct from other cell death pathways, such as apoptosis and necrosis. Consequently, citrullinated histone H3 (H3Cit) has been proposed as a specific biomarker for NET formation (7). In addition, Neutrophils produce NETs by expelling decondensed DNA chromatin coated with H3Cit, along with cytoplasmic granule enzymes such as myeloperoxidase (MPO) and neutrophil elastase, into the extracellular space (8). The soluble remnants of NETs, primarily cell-free (cf)-DNA, are found in supernatant fluid *in vitro* and in serum or tissue fluid *in vivo* (9).These structures play crucial roles in both immune response and the progression of neuroinflammation following stroke. NETs promote thrombosis and disrupt the blood-brain barrier, exacerbating neuroinflammatory responses. The disruption of the blood-brain barrier is critical as it allows the infiltration of peripheral immune cells, which further amplifies the inflammatory milieu within the central nervous system (10, 11).

Microglia, the resident immune cells of the central nervous system, play pivotal roles in mediating inflammatory processes following stroke. These cells can regulate inflammation through various mechanisms, including the release of pro-inflammatory cytokines, phagocytosis of damaged cells, and the modulation of other glial cell functions. The polarization of microglia, particularly toward M1 phenotype, is closely associated with inflammatory damage following stroke (12, 13). M1 microglia contribute to inflammation through the secretion of various cytokines, whereas M2 microglia play neuroprotective roles in stroke.

FK506 binding protein 5, known as FKBP5, belongs to the immunoaffinity protein family and plays a role in various cellular processes (14). As a chaperone protein, FKBP51 regulates the transcription factor nuclear factor kappa B (NF-κB), thereby influencing the expression of proinflammatory cytokines (15). Yu et al. found that FKBP5 is significantly upregulated in patients with acute ischemic stroke and the upregulation of FKBP5 in brain ischemia/reperfusion injury is associated with the severity of ischemic and reperfusion damage (16). Fang et al. reported that inhibiting the FKBP5-p38 MAPK axis attenuates oxygen-glucose deprivation/reperfusion-induced cardiomyocyte ferroptosis and excessive mitophagy, suggesting its cardioprotective potential against myocardial ischemia/reperfusion injury (17). A recent study demonstrated that the absence of FKBP5 decreases cell-crystal adhesion, diminishes apoptosis, enhances cell proliferation, suppresses M1 macrophage polarization and chemotaxis through inhibiting the NF-κB signaling (18). Our study observed the upregulation of FKBP5 in patients with ischemic stroke using transcriptome sequencing. However, whether FKBP5 regulates microglial polarization to influence ischemic stroke remains unknown.

Emerging research highlights the association between NETs and microglial activation in various neurological disorders, suggesting that understanding this interplay may yield valuable therapeutic insights (19, 20). Additionally, cytokines released by activated microglia may further promote NET formation, perpetuating a vicious cycle of inflammation (21). The relationship between FKBP5 and neuroinflammatory response also warrants further exploration, as FKBP5 has been implicated in regulating stress responses and immune function (22).

Thus, in this work, we hypothesized that FKBP5 might mediate ischemic stroke through regulating NETs and microglia polarization. To demonstrate this hypothesis, we first examined the pathological role of NETs in ischemic stroke through clinical cases and *in vivo* experiments, followed by *in vitro* investigations into FKBP5’s effects on NETs and microglial polarization along with the underlying mechanisms involved.

## Methods

### Ischemic stroke patients and samples

Patients admitted to Liaocheng People’s Hospital from January 2018 to September 2022, and diagnosed as ischemic stroke according to Chinese Guidelines for the Diagnosis and Treatment of Acute Ischemic Stroke 2018 were enrolled in this study. Inclusion Criteria: (1) Newly confirmed acute cerebral infarction via CT or MRI; (2) Age 50–70 years; (3) Time from symptom onset < 6 hours; (4) Neurological deficits (e.g., speech impairment or limb motor dysfunction) persisting for ≥ 30 minutes; (5) Ability to cooperate with study procedures, with both the patient and legally authorized representative providing signed informed consent after understanding the study; (6) National Institutes of Health Stroke Scale score ≥ 3 and ≤10. Exclusion Criteria: (1) Intracranial hemorrhage (including parenchymal, intraventricular, subarachnoid, or subdural/extradural hemorrhage); (2) History of severe head trauma or stroke within 3 months; (3) Major surgery within 4 weeks or arterial puncture at a non-compressible site within 1 week; (4) Chronic anticoagulant use with INR >1.7 or PT >15 seconds; (5) Abnormal procoagulant indices (e.g., platelet count <90 × 10^9^/L); (6) Severe uncontrolled hypertension, with ineffective pharmacological control; (7) Blood glucose <2.8 mmol/L or >22.2 mmol/L; (8) Radiological evidence of large territory infarction (>1/3 of MCA territory on CT/MRI). For comorbidities, calcium channel blockers or angiotensin-converting enzyme inhibitors were generally selected for hypertension management, while biguanides, insulin secretagogues, or subcutaneous insulin administration were typically employed for glycemic control. Through calculation, a total of 80 samples were gathered, consisting of 40 from ischemic stroke patients and 40 from age- and sex-matched healthy donors (HDs). Cells in the blood samples were eliminated through low-speed centrifugation, and plasma was collected and stored at -80°C. In addition, ten brain tissues from patients with cerebral infarction were collected to validate the expression of FKBP5 detected using transcriptome sequencing previously, and brain tissue obtained from individuals who died in car accidents was used as the control group. The study received approval from the ethics committee of Liaocheng People’s Hospital and adhered to the principles of the Declaration of Helsinki. Informed consent was obtained from all participants, and written consent was specifically obtained from the parents of participants.

### H3cit measurement

Plasma samples were diluted (1:2), and the levels of H3cit were measured according to the manufacturer’s instructions using the Citrullinated Histone H3 ELISA Kit (YJ701102, mlbio).

### Measurement of MPO-DNA complexes

MPO-DNA complexes were quantified using an ELISA kit (YJ360097, mlbio). Briefly, a 96-well plate was coated overnight with anti-MPO antibody at 4°C, followed by blocking with 2.5% BSA in PBS for 2 hours at room temperature. After washing, the plate was incubated for 90 minutes at room temperature with 20% human or mouse plasma in blocking buffer. Following five washes, the plate was incubated for another 90 minutes at room temperature with anti-DNA antibody. After five additional washes, the plate was developed with ABTS substrate.

### Quantification of plasma DNA

Plasma samples were collected from human whole blood by centrifugation at 500×g for 20 minutes. The DNA in plasma was quantified according to the manufacturer’s instructions of the Quant-iT PicoGreen dsDNA Assay kit (12641ES01, Yeasen).

### tMCAO model

A transient middle cerebral artery occlusion (tMCAO) stroke model was established as previously reported. Briefly, the right middle cerebral artery (MCA) was occluded by inserting a standardized monofilament (Doccol Corp.) through the right internal carotid artery. The occlusion was maintained for 60 minutes. The occurrence of ischemic stroke was confirmed by neurological tests during the occlusion period. Anesthesia was induced using the inhalation of 5% isoflurane and maintaining with 2% isoflurane. Buprenorphine was administered 1 hour before surgery and every 12 hours as needed. A sham surgery was performed similarly, without the monofilament insertion. Mice were maintained under standard housing conditions with ad libitum access to food and water, and a controlled 12-hour light/12-hour dark photoperiod cycle. Mice were excluded from endpoint analyses based on the following criteria: (a) death within 12 hours after tMCAO; (b) an operation time longer than 10 minutes; (c) the occurrence of surgical complications. Mice brains were visually inspected for surgical complications and stained with 2,3,5-triphenyl tetrazolium chloride (TTC) to confirm the ischemia-related mortality.

### Animal experimental design

To elucidate therapeutic effect of CI-amidine, an inhibitor of PAD4 that can inhibit the formation of NETs (23), on stroke, a total of 45 male C57BL/6 mice (6–8 weeks old) were randomly divided into three groups (n=15/group): the sham group, tMCAO model group (stroke) and tMCAO+CI-amidine group (stroke+CI). CI-amidine (1373232-26-8, MCE) was dissolved in saline and injected 1 hour after tMCAO induction. The compound was administered once daily at a dose of 50 mg/kg via intraperitoneal injection for three consecutive days. All subjects were euthanized 24 hours after the final administration for subsequent endpoint analyses. The animal study was reviewed and approved by the Ethics Committee for Experimental Animals of Liaocheng People’s Hospital.

### Determination of brain water content

Cerebral edema induced by elevated brain water content constitutes a cardinal pathological process that severely compromises prognosis following ischemic stroke (24). Thus, we detected the brain water content in the mice to evaluate the condition of stroke (25). Mice were decapitated under deep anesthesia 24 hours after tMCAO induction. Brain samples were quickly excised and divided into left and right cerebral hemispheres, cerebellum and brain stem. These four parts were weighed (wet weight), then dried at 55°C for 72 hours and weighed again (dry weight). The BWC percentage was calculated using the formula: water content %= (wet weight−dry weight)/wet weight×100%.

### Neurons isolation

Primary neurons were prepared from the brain tissue of 6-8-week-old C57BL/6 mice. Dissociated cortical cells were plated onto poly-D-lysine-coated 6-well plates at a density of 7×10^5 cells per well. After 4 hours of plating, the culture medium was replaced with Neurobasal medium supplemented with B-27. Subsequently, cells were maintained in a humidified incubator at 37°C with 5% CO_2_.

### MACS sorting of microglia

Microglia isolation was performed using CD11b-based MACS sorting. After tissue dissociation, cells were co-stained with Anti-CD11b Magnetic Microbeads (Miltenyi Biotec, CD11b MicroBeads Ultrapure) for 15 minutes on ice under light-protected conditions. Cells were subsequently washed with PBS and centrifuged at 400 ×g for 10 minutes at 4°C. The supernatant was aspirated, and cells were resuspended in sorting buffer. Prior to separation, LS columns were pre-equilibrated with 3 ml PBS and positioned in a magnetic separator. The cell suspension was applied to the column, followed by three washes with 10 ml PBS each. Magnetically labeled cells were eluted by removing the column from the separator and flushing with 5 ml PBS into a 15 ml collection tube. The isolated microglia were pelleted by centrifugation (400 ×g, 10 min) and collected for downstream applications.

### Hematoxylin-eosin staining

HE staining was performed to evaluate morphological changes following tMCAO as previous study (26). Brain tissues with infarction from each group were collected, fixed in paraformaldehyde, dehydrated, embedded in wax, and sectioned. The sections were subsequently stained with HE as previously reported.

### Immunohistochemistry analysis

As previous study (27), Paraffin-embedded brain tissue sections from each group were deparaffinized by baking at 60°C for 1 hour, followed by the treatment with xylene and a gradient of ethanol for rehydration. Antigen retrieval was performed using high-pressure treatment with citrate buffer. After quenching endogenous peroxidase and blocking with 5% goat serum, sections were incubated overnight at 4°C with primary antibodies (H3cit, bsm-33042, Bioss; MPO, ab208670, Abcam). Subsequently, goat anti-rabbit secondary antibodies were applied at 37°C for 1 hour. DAB staining was conducted for 5–10 minutes, followed by hematoxylin counterstaining for 20 seconds. Finally, the slides were dehydrated, sealed, and observed under a microscope.

### Immunofluorescence staining

Mice brain tissues with infarction were perfused with ice-cold PBS and swiftly extracted. The brains were fixed in 4% paraformaldehyde (PFA) at 4°C for 24 hours. Coronal sections of 8 μm thickness were obtained using a cryostat (Leica, Model CM1950, Germany). The sections were washed with PBS and permeabilized with 0.1% Triton X-100 (Sigma Aldrich) for 30 minutes. After permeabilization, the sections were incubated with 3% BSA at room temperature for 1 hour to block non-specific binding. Subsequently, the sections were incubated overnight at 4°C with following primary antibodies: MPO (ab208670, Abcam). The sections were then incubated with species-appropriate Alexa Fluor-conjugated IgG (1:500, Invitrogen, USA) for 1 hour at room temperature. Nuclei were counterstained using DAPI before imaging.

### Flow cytometry analysis

Mouse neuronal cells isolated from brain tissue were resuspended in binding buffer and dual-labeled with Annexin V-FITC and propidium iodide reagent (Beyotime) for 20 minutes without light. The apoptotic rate was determined by the flow cytometer (Beckman Coulter, Brea, CA, USA). The apoptotic rate is calculated as the sum of cells in Q2 (Upper Right quadrant) and Q3 (Lower Right quadrant), where Q2 represents late apoptotic cells and Q3 represents early apoptotic cells. Three independent replicates were conducted, each using cells isolated from separate mice.

### TUNEL staining

To observe apoptotic neurons, TUNEL staining (red) was conducted using a TUNEL kit (Beyotime) according to the manufacturer’s instructions. In brief, neuronal cells on coverslips were fixed with 4% paraformaldehyde for 15 minutes, rinsed three times (3 minutes each) with PBST, then permeabilized with 0.2% Triton X-100 in 1× PBS at room temperature for 5 minutes. After three additional PBS washes, samples were equilibrated with 1× Equilibration Buffer for 15 minutes followed by three PBS rinses. The reaction working solution was applied after buffer removal and incubated at 37°C for 60 minutes. DAPI was added for nuclear counterstaining (5 minutes, protected from light), with excess dye removed by PBS washing. Antifade mounting medium was applied to seal coverslips while avoiding bubble formation. Finally, samples were visualized and representative images captured using fluorescence microscopy (Olympus, Japan).

### Cell culture and transfection

BV2 microglial cell line was cultured in a humidified atmosphere with 5% CO_2_ at 37°C in complete DMEM medium supplemented with 10% fetal bovine serum (FBS) and 1% penicillin-streptomycin. Upon reaching 70-80% confluence, cells were transfected using Lipofectamine™ 2000 (Invitrogen; Thermo Fisher Scientific, Inc) according to the manufacturer’s instructions. For neutrophils co-culture, culture supernatants of BV2 microglial cell were collected 48 h post-transfection and applied to isolated neutrophils. To investigate the functional role of FKBP5, BV2 cells were divided into four groups: Oe-NC (overexpression control), Oe-FKBP5 (FKBP5 overexpression), si-NC (silencing control) and si-FKBP5 (FKBP5 silencing).

### Quantitative real-time polymerase chain reaction

Total RNA was extracted from the cells using Trizol reagent (Invitrogen, Thermo Fisher Scientific), and cDNA was synthesized from 2 μg RNA using the PrimeScript RT Reagent Kit (Takara Bio, Tokyo, Japan) following the manufacturer’s instructions. The PCR assays were performed on a Real-Time PCR Detection System (Bio-Rad, Hercules, CA, USA) using SYBR Green PCR Master Mix (Applied Biosystems, Waltham, MA, USA). GAPDH was served as an internal control. mRNA expression was calculated using the comparative 2^−ΔΔCt^ method. The primers used in this study were listed in **Table 1**.

**Table 1:** Primer sequence details.

Gene ID	Primer sequence (5’-3’)
FKBP5	F-CGGCGACAGGTTCTCTACTTA
	R-CCCTGCCTCTCCAAAACCAT
CCL5	F-TCATTGCTACTGCCCTCTGC
	R-TCGGGTGACAAAGACGACTG
GAPDH	F-GCACCGTCAAGGCTGAGAAC
	R-AGGTGACCGCAGAAGTGGT

F, Forward; R, Reverse.

### Western blot analysis

Protein samples were prepared with an equal volume of loading buffer and denatured at 100°C for 15 minutes. 20 μg of protein in each sample was separated using SDS-PAGE and subsequently transferred to PVDF membranes (Millipore, Temecula, CA, USA). Membranes were blocked with non-specific blocking solution and then incubated overnight at 4°C with primary antibodies: FKBP5 (14155-1-AP, Proteintech), CCL5 (AF5151, Affinity), ERK (BF8004, Affinity), p-ERK (AF1015, Affinity), P38 (14064-1-AP, Proteintech), p-P38 (28796-1-AP, Proteintech), p-JNK (60666-1-Ig, Proteintech), JNK (66210-1-IG, Proteintech), CD206 (60143-1-IG, Proteintech), and GAPDH (60004-1-Ig, Proteintech). After washing in TBST, membranes were incubated with HRP-conjugated secondary antibodies (1:5000, Cell Signaling Technology, USA) at room temperature for 1 hour. GAPDH served as a loading control. Protein bands were visualized using an imaging system (Bio-Rad, Hercules, CA, USA) and quantified using ImageJ software (Version 1.46r, Wayne Rasband, USA). Protein expression levels were quantified by normalizing the grayscale values of target bands to GAPDH loading controls.

### Cytokine analysis

Serum levels of IL-1β (ml098416, mlbio), IL-10 (mIC50274-1, mlbio), and TNFα (mIC50536-1, mlbio), iNOS (mlswE3080, mlbio) were determined using ELISA kits (Neobioscience, Beijing, China) according to the manufacturer’s instructions. The absorbance measurements were recorded at 450 nm using a Bio-Tek microplate reader, and the corresponding concentration was converted through the standard curve. The assays were conducted to assess the release of inflammatory cytokines from serum samples.

### Neutrophil isolation

Neutrophils were isolated from the circulation using Percoll (GE Healthcare) gradient separation (28). First prepare 75% and 62% Percoll solutions. Dilute 5 mL peripheral blood 1:1 with serum-free RPMI 1640. In a 50 mL tube, layer 10 mL 75% Percoll, then carefully add 10 mL 62% Percoll on top. Slowly add 20 mL diluted blood. Centrifuge at 200×g (25 min) then 400×g (15 min) with no brake. Collect neutrophils from the 75%-62% interface, wash with RPMI 1640 (300×g, 5 min), and resuspend in 1 mL neutrophil buffer.

### Co-immunoprecipitation

Protein stock was combined with 4× Protein SDS PAGE Loading Buffer, diluted to 1 mg/mL, and denatured at 100°C for 5 minutes to serve as an input control. For Co-IP, 200 μg of protein lysate supernatant was mixed with 2 μg of FKBP5 or CCL5 antibody at 4°C for antigen-antibody binding. A negative control involved using normal rabbit IgG in place of the FKBP5 antibody, following the same incubation period of 24 hours. Dynabeads were pretreated with the lysate in an ice bath three times, at 10-minute intervals, and collected using a magnetic stand. The antigen-antibody mixture was added to the pretreated Dynabeads and incubated for another 2 hours at 4°C with continuous rotation, forming the antigen-FKBP5/CCL5 antibody-Dynabead complex. The beads were magnetically separated, discarding unbound proteins, and mixed with 30 μL of 1× Protein SDS-PAGE loading buffer. The complex was then denatured at 100°C for 5 minutes, with the supernatant collected as the immunoprecipitated protein sample. Finally, western blot analysis was conducted to verify the interactions among FKBP5 and CCL5.

### Statistical analysis

GraphPad Prism software version 9.0 was used for data analysis. Data were presented as the mean ± standard deviation. Spearman correlation analysis was used to investigate whether markers of NET formation correlated with ischemic stroke outcomes, with 0.7 ≤ r ≤ 1 termed as strong correlation, 0.4 ≤ r < 0.7 termed as moderate correlation, and 0.2 ≤ r < 0.4 termed as weak correlation (29). For comparisons across multiple groups, the one-way analysis of variance (ANOVA) followed by Tukey ‘s honest significant difference test was used. To assess differences between two specific groups, the unpaired t-test was employed. Normality testing was performed for clinical samples, and parametric tests were selected when the data met assumptions of normality and homogeneity of variance. A p-value of less than 0.05 was considered to indicate statistical significance.

## Results

### Increased expression of FKBP5 and NET in patients with ischemic stroke

**Figure 1:**
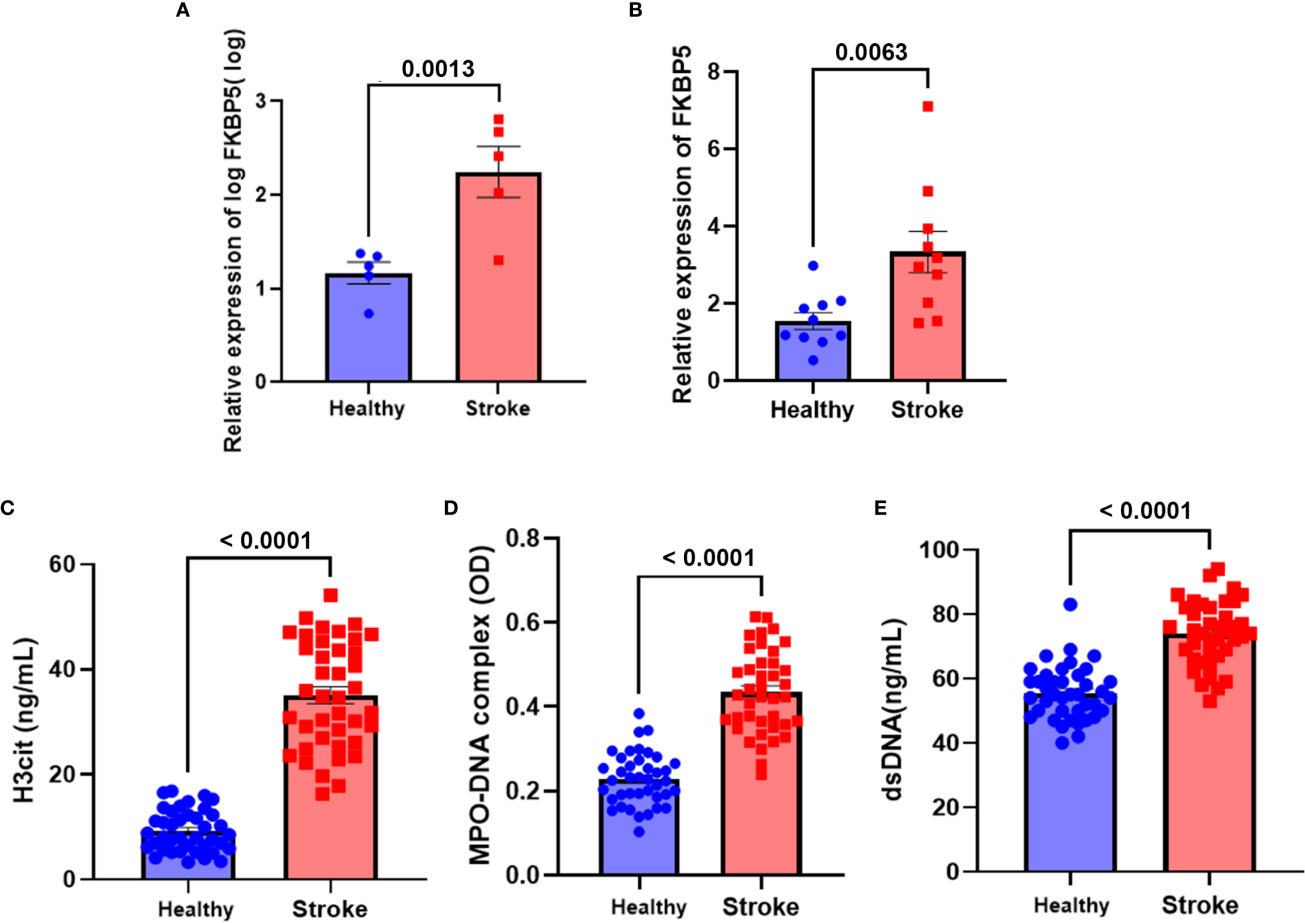
Increased expression of FKBP5 and NET markers in ischemic stroke patients. Plasma samples and brain tissues were collected within 24 hours from ischemic stroke patients and healthy donors (HDs). **(A)** FKBP5 is significantly upregulated in the brain tissues in stroke group, a visualization of the sequencing results, n = 5. **(B)** FKBP5 is significantly upregulated in the brain tissues of expanded cohort in stroke group (n = 10). Levels of H3cit **(C)** and MPO-DNA complexes **(D)** in plasma were quantified using ELISA. **(E)** dsDNA levels in plasma were assessed using the PicoGreen dsDNA Assay kit. n = 40 per group.

Peripheral blood from 40 patients with ischemic stroke and 40 HDs were collected. Clinical characteristics were summarized in **Table 2**. Patients had a mean age of 62 years, with 18 males (45.0%); comorbid diabetes was present in 42.5% (n=17). Brain tissues were extracted from patients with cerebral infarction and individuals who died in car accidents (each comprising 5 individuals). Transcriptome sequencing data revealed that FKBP5 is significantly upregulated in the patient group, with the log2 fold being changed of 5.405 (p = 1.82E-13), as shown in **Supplementary Tables S1, S2**. The visualization of sequencing results was shown in **Figure 1A**. Expanded clinical samples further confirmed the high expression of FKBP5 in stroke patients (**Figure 1B**). Expression levels of NET-associated markers (H3cit, MPO and dsDNA) were measured in the serum of stroke patients. Our results found that the expression of the NET marker H3cit was significantly increased in ischemic stroke patients within 24 hours compared to HDs (**Figure 1C**). ELISA analysis demonstrated a marked elevation in the total content of the MPO-DNA complex in plasma samples from stroke patients compared to those from HD (**Figure 1D**). Additionally, plasma DNA levels were significantly increased in stroke patients in comparison to the HDs (**Figure 1E**). The results indicated the elevated NET formation in the peripheral blood of stroke patients compared to those of HD. We next used a Spearman correlation analysis to investigate whether markers of NET formation correlated with ischemic stroke outcomes. As shown in **Table 3**, both plasma H3cit and MPO-DNA levels positively correlated with stroke severity (r = 0.332, P = 0.036 and r = 0.391, P = 0.013, respectively) and stroke outcomes (r = 0.379, P = 0.016 and r = 0.466, P = 0.002, respectively), implying the positive correlation between NET formation and stroke severity.

**Table 2:** Clinical characteristics of the stroke patients and healthy donors.

Characteristics	Healthy donors (n=40)	Ischemic stroke (n=40)	P-value
Age (mean ± SD; years)	57 ± 15	62 ± 14	0.1273
Female (%)	40%	45%	0.7979
Diabetes (%)	8%	42.50%	0.0003
Hyperlipidemia (%)	5.00%	22.50%	0.0239
Hypertension (%)	12.50%	57.50%	<0.0001
Hyperhomocysteinemia (%)	5%	38%	0.0004
Stroke Severity (median(range); NIHSS at admission)		5 (0-15)	
Stroke Outcome (median(range); mRS at discharge)		2 (0-5)	
TOAST subtype			
LAA		11	NA
CE		6	NA
SVD		12	NA
Other		5	NA
Unknown		6	NA
Medication			
Anti-coagulation		13	NA
Anti-platelets		16	NA
Thrombolysis		11	NA

SD, Standard deviation; NIHSS, National Institutes of Health Stroke Scale; TOAST, Trial of Org 10172 in Acute Stroke Treatment; LAA, Large artery atherosclerosis; CE, Cardioembolic; SVD, Small vessel disease.

**Table 3:** Correlations between NET markers, stroke severity and stroke outcome.

NET markers	Stroke severity	Stroke outcome
H3cit	r = 0.3322; p = 0.0362	r = 0.3788; p =0.0159
MPO-DNA	r = 0.3908; p = 0.0127	r = 0.4663; p = 0.0024
dsDNA	r = -0.03796; p = 0.8161	r = -0.1895; p = 0.2416

### Inhibition of NETs attenuates brain injury in tMCAO model

**Figure 2:**
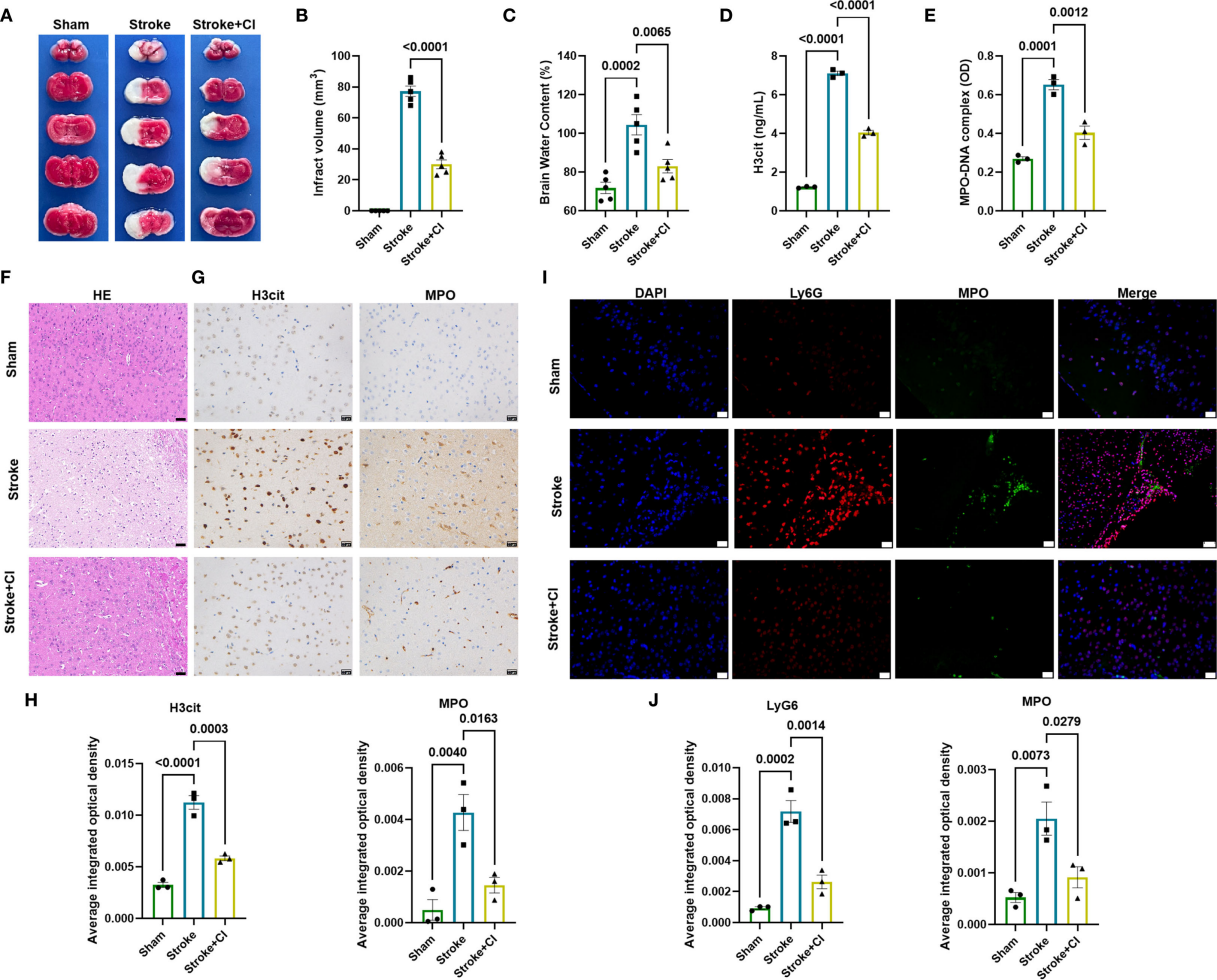
Inhibition of NETs attenuates brain injury in tMCAO model. **(A)** Coronal brain sections stained with TTC 24 hours after tMCAO, the white areas represent the infarct regions. Dot plot showing infarct volume **(B)** and brain water content **(C)** in each group, n = 5. Levels of H3cit **(D)** and MPO-DNA complexes **(E)** were measured using ELISA 24 hours post-stroke induction, n = 3. **(F)** HE staining showing neuronal morphological changes in each group. Scale bar indicates 50μm. **(G, H)** Immunohistochemical staining showing positive expression for H3cit and MPO in each group (Brown staining indicates positive expression). Scale bar indicates 20 μm, n = 3. **(I, J)** Representative immunofuorescence micrographs of MPO cells (green) with Ly6G (red) in each group. Scale bar indicates 20μm, n = 3. The NET inhibitor is CI-amidine, abbreviated as CI.

We then intended to demonstrate if the administration of the NETs inhibitor leads to the improvement of stroke-related brain injury in tMCAO mice, as well as changes in NETs-related indicators. Mice were randomly divided into three groups: sham group, tMCAO model (stroke) and tMCAO+CI-amidine (stroke+CI). Our results demonstrated that the administration of the NETs inhibitor significantly reduced the infarct area and water content, a factor that constitutes a cardinal pathological process that severely compromises prognosis following ischemic stroke, in the brains of mice subjected to stroke by TTC staining and BWC analysis (**Figures 2A–C**), demonstrating the relieving effect of NET inhibition on stroke. Plasma levels of H3cit and MPO-DNA complexes were increased 24 hours post-stroke induction and decreased following the treatment of NETs inhibitor (**Figures 2D, E**). Histological examination of the peri-infarct cortex using HE staining revealed a reduction in neuronal damage following NETs inhibitor treatment (**Figure 2F**), further indicated the relieving effect of NET inhibition on stroke. Immunohistochemical analysis showed a higher positive expression for H3cit and MPO in the stroke group compared to the sham group, while the positive expression was decreased in the stroke+CI group (**Figures 2G, H**). Finally, immunofluorescence staining showed a higher positivity rate for Ly6G (neutrophil marker) and MPO in the stroke group compared to the sham group, while the positivity rate was decreased in the stroke+CI group (**Figures 2I, J**). These results further confirmed the elevated NET formation in stroke.

### Effects of NET inhibitor on neuronal apoptosis

**Figure 3:**
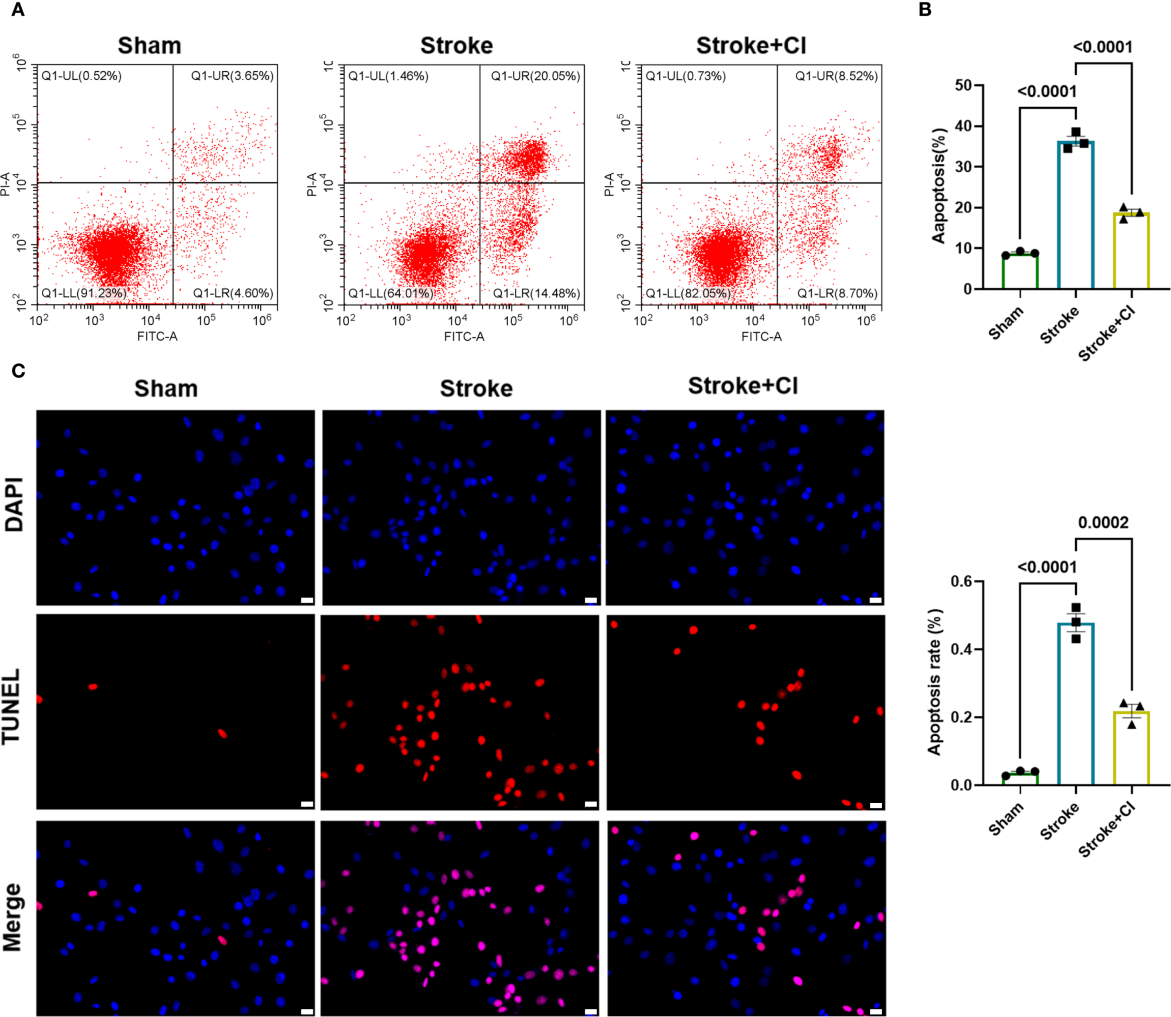
Effects of NET inhibitor on neuronal apoptosis. Primary neuronal cells were isolated from mouse brain tissue and analyzed for apoptosis using flow cytometry **(A, B)** and TUNEL (red) staining **(C)**. The NET inhibitor is CI-amidine, abbreviated as CI. n = 3.

Based on the results of histological examination, we further evaluated the effects of NET inhibitor on neuronal apoptosis. Primary neurons were isolated from mouse brain tissue. Flow cytometry and TUNEL assays were used to evaluate the effect of NETs inhibitors on neuronal apoptosis. Compared to the sham group, the stroke group exhibited significantly increased levels of neuronal apoptosis, which were reduced after NETs inhibitor treatment (**Figures 3A, B**). The TUNEL assay produced consistent results, showing that the number of apoptotic cells in the stroke group was higher compared to the sham group, while the administration of the inhibitor resulted in a decrease in the number of apoptotic cells (**Figure 3C**).

### Effects of FKBP5 expression on microglial polarization and NET formation

**Figure 4:**
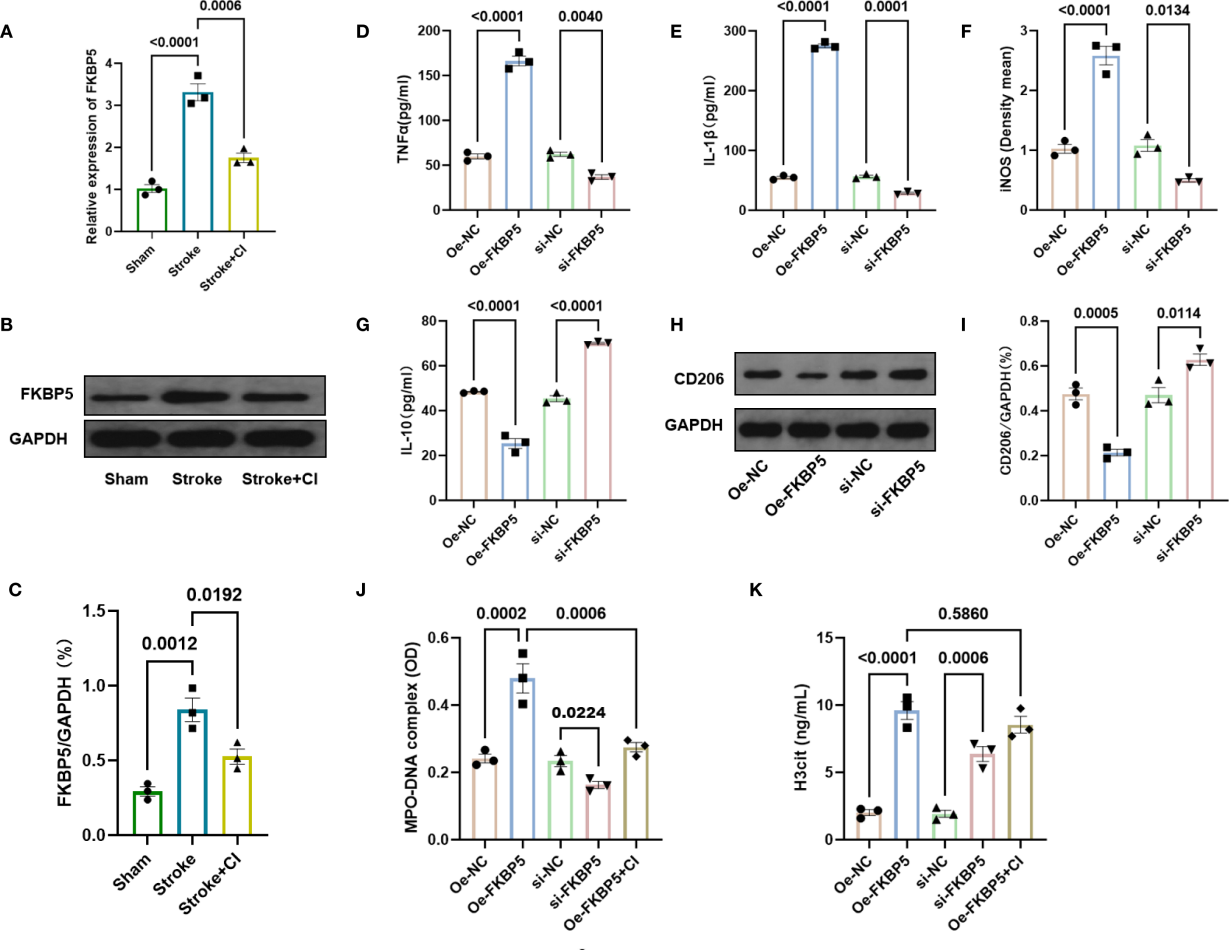
Effects of FKBP5 expression on microglial polarization and NET formation. **(A)** Relative expression of FKBP5 in primary microglia by qPCR. Representative western blotting images **(B)** and quantitative analysis **(C)** of FKBP5 in primary microglia. Levels of TNFα **(D)**, IL-1β **(E)**, iNOS **(F)** and IL-10 **(G)** were quantified using ELISA in different groups. Representative western blotting images **(H)** and quantitative analysis **(I)** of CD206 in different groups. ELISA results of MPO-DNA complexes **(J)** and H3cit **(K)** for co-culture of BV2 cells with neutrophils from the peripheral blood of patients. n = 3.

To investigate the function of FKBP5 in microglial polarization, primary microglia were isolated from brain tissue, and qPCR and Western blotting analyses were conducted to assess FKBP5 expression levels. The results showed that FKBP5 level was significantly elevated in the stroke group compared to the sham group, and the expression significantly decreased in the stroke+CI group (**Figures 4A–C**). Furthermore, BV2 microglial cells were transfected with FKBP5 overexpression and knockdown vectors, showing that FKBP5 overexpression promoted M1 polarization, evidenced by elevated expression of M1 markers TNFα, IL-1β and iNOS (**Figures 4D–F**). In contrast, the expression of M2 markers IL-10 and CD206 decreased when FKBP5 was overexpressed (**Figures 4G–I**). Co-culture of BV2 cells with neutrophils from peripheral blood of patients revealed that FKBP5 overexpression significantly increased MPO-DNA and H3cit levels, promoting NET formation, while NET inhibitor treatment decreased the levels of MPO-DNA and H3cit (**Figures 4J, K**). Collectively, these results suggested that FKBP5 facilitates M1 polarization of microglia and promotes the formation of NETs.

### Interaction between FKBP5 and CCL5

**Figure 5:**
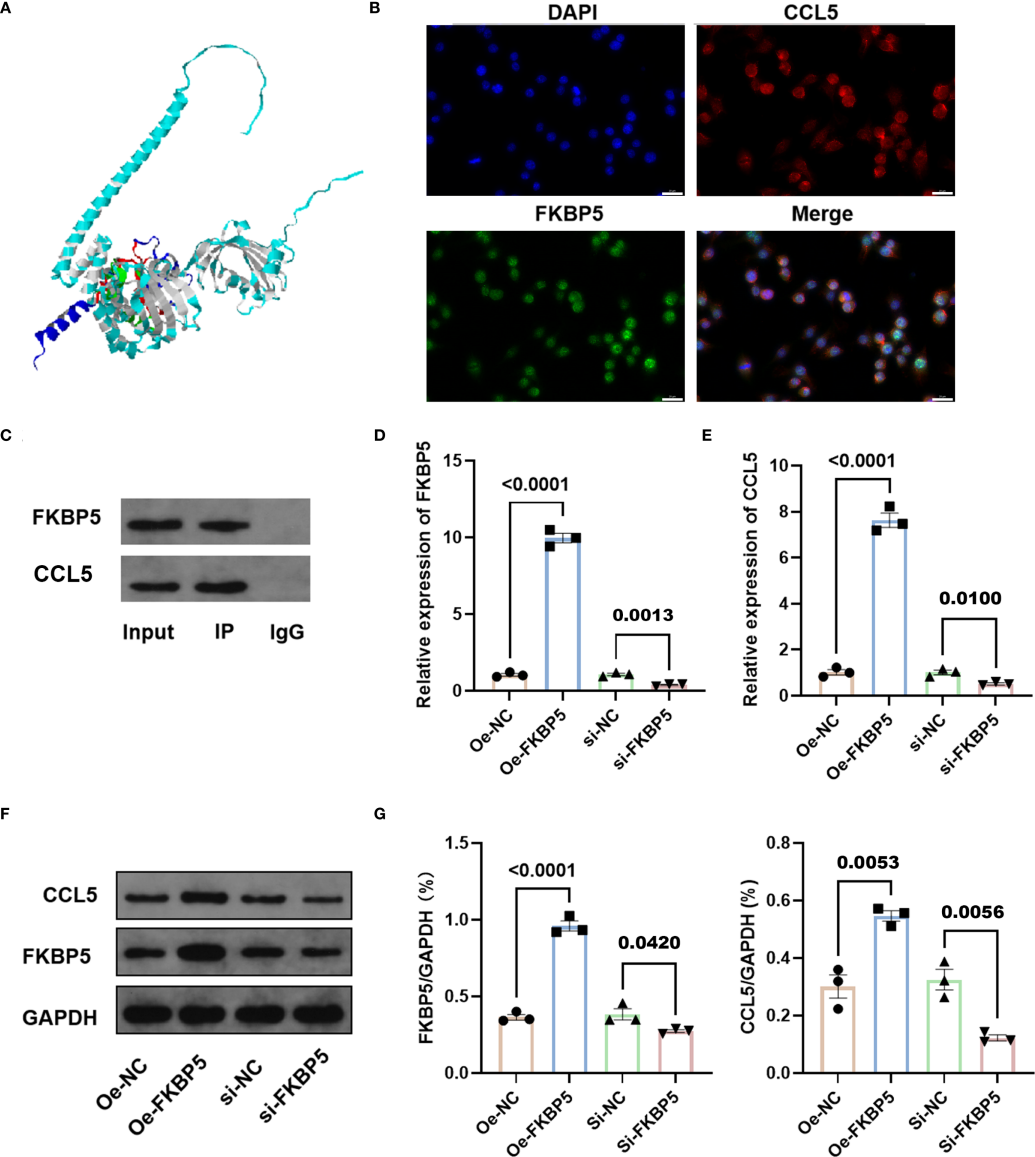
Interaction between FKBP5 and CCL5. **(A)** Visualization of the rigid molecular simulations showing the docking interaction between FKBP5 and CCL5. The docking results indicate that the free energy is below zero, suggesting spontaneous docking. CCL5 is represented in blue and FKBP5 in cyan, while the regions of interaction are highlighted in red and green. **(B)** Immunofluorescence staining of CCL5 (red) and FKBP5 (green) within the cells. The overlap in fluorescence signals indicates areas where both proteins are present together. Scale bar indicates 20μm. **(C)** Western blot analysis to detect FKBP5 in protein-antibody complexes purified from Co-IP assays. Relative expression of FKBP5 **(D)** and CCL5 **(E)** in BV2 cells by qPCR in different groups. Representative western blotting images **(F)** and quantitative analysis **(G)** of FKBP5 (left panel) and CCL5 (right panel) in BV2 cells. n = 3.

One recent study indicated that the level of CCL5 may be predictive of infarct volume outcomes in patients with ischemic stroke (30). To explore the potential interaction between FKBP5 and CCL5, we conducted the molecular simulation docking visualization analysis using human proteins (**Figures 5A**). The rigid docking between these proteins yielded a calculated free energy of −8.2 kcal/mol (a negative binding energy value), which implies a structural foundation for spontaneous docking between two predicted protein models. Moreover, subcellular localization analysis suggested a potential overlap during the entirety of cellular development (**Figures 5B**). To further investigate the molecular association between FKBP5 and CCL5, the Co-IP assay was performed (**Figures 5C**) and our results confirmed that CCL5 and FKBP5 can bind to one another within total protein extracts. Further analysis via qPCR and Western blotting confirmed that FKBP5 promotes the expression of CCL5 following transfection of overexpression and interference vectors of FKBP5 into BV2 cells (**Figures 5D–G**). These results revealed the regulatory effect of FKBP5 on CCL5.

### FKBP5 activates the MAPK signaling pathway via CCL5

**Figure 6:**
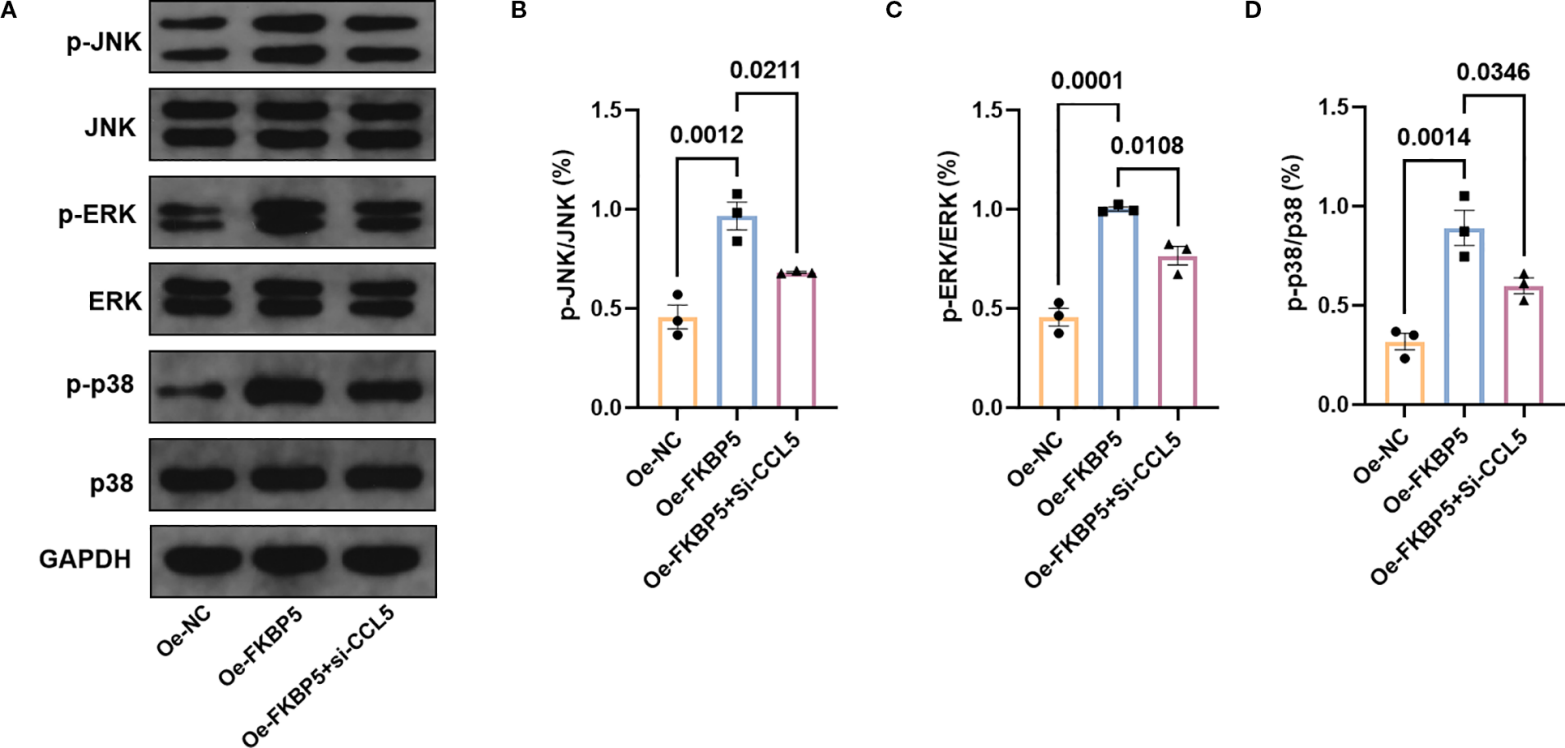
FKBP5 activates the p38 MAPK signaling pathway via CCL5. **(A)** Western blot analysis of proteins in MAPK signaling pathway in different groups. Grayscale quantification of western blot analysis of p-JNK **(B)**, p-ERK **(C)**, and p-p38 **(D)** in different groups. n = 3.

Pathway enrichment analysis of differentially expressed gene between ischemic stroke patients and HDs revealed that the MAPK signaling pathway may play an important role in stroke (**Supplementary Table S3**). Further validation experiment demonstrated that the overexpression of FKBP5 can activate the MAPK signaling pathway, leading to enhanced expression levels of p-p38, p-JNK and p-ERK. When BV2 cells were co-transfected with the FKBP5 overexpression vector (Oe-FKBP5) and CCL5 interference vector (si-CCL5), it was observed that the promoting effect of FKBP5 on the MAPK signaling pathway could be inhibited by si-CCL5 (**Figure 6A**). The grayscale analysis of the proteins supported these findings (**Figures 6B–D**).

### The effect of FKBP5-CCL5 on BV2 cell polarization and neutrophil NETs formation via the MAPK signaling pathway

**Figure 7:**
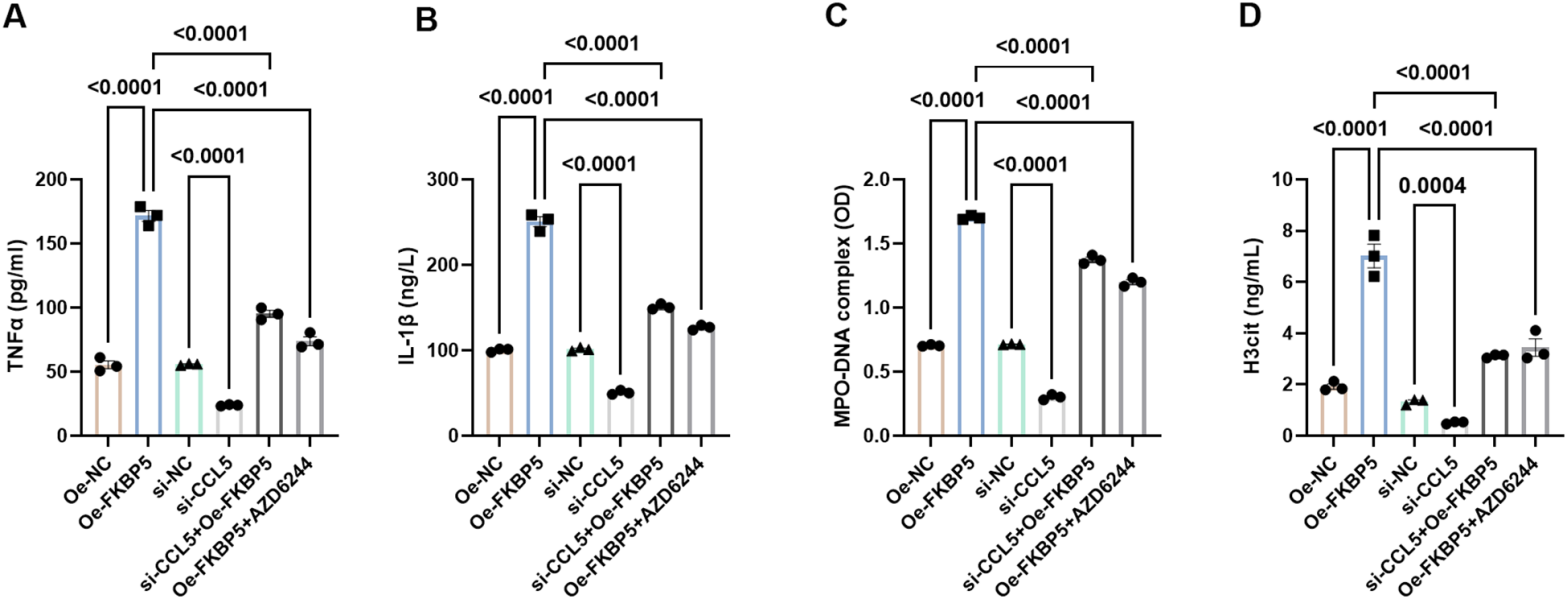
Influence of FKBP5 on BV2 cell polarization and neutrophil NETs formation via the p38 MAPK signaling pathway. Levels of pro-inflammatory cytokines, TNFα **(A)** and IL-1β **(B)**, were quantified using ELISA in different groups. Levels of NET markers, MPO-DNA **(C)** and H3cit **(D)**, were quantified using ELISA in different groups. AZD6244 is an inhibitor of the MAPK pathway. n = 3.

Previous studies have identified the impact of MAPK signaling pathway on NETs formation and cell polarization (31, 32). Based on the above findings, we hypothesize that FKBP5-CCL5 -mediated regulation of microglial polarization and NET formation may be associated with the MAPK signaling pathway. ELISA assays revealed that the levels of pro-inflammatory cytokines TNFα and IL-1β are significantly increased following the overexpression of FKBP5 (**Figures 7A, B**). Treatment with the MAPK pathway inhibitor AZD6244 (10 μM) or si-CCL5 led to a marked reduction in the expression of TNFα and IL-1β (**Figures 7A, B**). Additionally, when BV2 cells overexpressing FKBP5 were co-cultured with neutrophils following AZD6244 treatment or si-CCL5, it was observed that AZD6244 or si-CCL5 effectively inhibited the increase of NET markers (MPO-DNA and H3cit) induced by FKBP5 (**Figures 7C, D**). Overall, these results confirmed the involvement of the MAPK pathway in mediating the effects of FKBP5-CCL5.

## Discussion

This work validated our hypothesis that FKBP5 might mediate ischemic stroke through regulating NETs and microglia polarization. The current findings demonstrate that FKBP5, through its interaction with CCL5, activates the p38 MAPK signaling pathway in BV2 cells. This activation promotes the differentiation of BV2 cells into M1 macrophages, resulting in the release of pro-inflammatory factors such as IL-1β and TNF-α. In addition, this signaling cascade enhances the formation of NETs, which contributes to subsequent neurodegeneration and the apoptosis of neuronal cells.

NETs - lattices of extracellular DNA released by neutrophils - function to entrap and eliminate pathogens (33). In the bloodstream, NETs promote thrombus formation to restrict dissemination of infectious agents (34). However, excessive NET generation exacerbates cardiovascular pathology through clot stabilization (35), microvascular thrombosis induction (36), and endothelial cell death (37). Multiple studies have identified NETs within thrombi from ischemic stroke patients, where they confer resistance to tissue-type plasminogen activator (35, 38, 39). Herein, we demonstrate the presence of NETs in patients with ischemic stroke, as demonstrated by the upregulation of NET formation markers (H3cit, MPO, and dsDNA). This was further observed in tMCAO stroke model mice, and NET inhibition suppressed neurons apoptosis isolated from tMCAO stroke model mice. Similar to previous study (40), these findings established NET formation as a clinically significant pathogenic mechanism in ischemic stroke. Further *in vitro* experiments demonstrated the promotion impact of FKBP5 on NET formation, indicating the potential function of FKBP5 in stroke through regulation NET.

The current findings also suggested that FKBP5 overexpression promoted microglia M1 polarization, evidenced by elevated expression of M1 markers TNFα, IL-1β and iNOS, and inhibited M1 polarization, evidenced by attenuated expression of M2 markers IL-10 and CD206 (41). Microglia, the resident immune cells of the central nervous system, are established mediators of neuroinflammation in ischemic stroke (42, 43). Following activation, these cells undergo polarization into pro-inflammatory (M1) or anti-inflammatory (M2) phenotypes, exerting dual-and often opposing-effects on post-ischemic brain injury, repair, and regeneration (44). M1-polarized microglia release proinflammatory cytokines (e.g., TNFα, IL-1β) and chemokines that amplify neuroimmune responses and exacerbate neuronal damage. Conversely, M2 microglia promote tissue repair through secretion of anti-inflammatory mediators (e.g., IL-10, TGF-β) that resolve inflammation and support neurorestorative processes (13, 44). Collectively, this study indicated that FKBP5 might mediate ischemic stroke through regulating microglia polarization. In addition, the release of these pro-inflammatory cytokines, such as IL-1β and TNFα, is instrumental in enhancing the formation of NETs. The increased presence of NETs promotes neuroinflammation and contributes to the formation of thrombi, which can obstruct cerebral blood flow and worsen ischemic conditions (11). The subsequent neurodegeneration and apoptosis of neuronal cells can be attributed to sustained inflammatory environment fostered by NETs and the activated p38 MAPK pathway (45). This is significant because the continuous cycle of inflammation and cell damage leads to detrimental outcomes in stroke patients, emphasizing the need for therapeutic approaches that can disrupt this cycle. Given the dual role of NETs in promoting thrombosis and neuroinflammation, targeting NET formation (CI-amidine utilization) or promoting their clearance represents a promising therapeutic strategy.

The interplay between FKBP5 and CCL5 is particularly noteworthy, as CCL5 is a chemokine that attracts immune cells to sites of injury (46). The interaction between FKBP5 and CCL5 further underscores the role of FKBP5 in mediating inflammatory signals within the central nervous system. We propose that FKBP5 not only initiates the inflammatory cascade but also serves to amplify it, consequently leading to the promotion of neuronal cell apoptosis and neurodegeneration. As transcriptome sequencing predicated the important function of MAPK pathway in stroke, and previous study has reported that CCL5 can directly initiate M1 polarization and inhibit M2 polarization through the activation of the MAPK pathway via CCR1 and CCR5 receptors (47). Thus, we therefore prioritized analysis of the MAPK signaling cascade based on preliminary evidence. Intriguingly, FKBP5 exhibited MAPK pathway-activating functionality, which was suppressed upon CCL5 inhibition co-treatment. MAPKs are a crucial family of proteins that play significant roles in various cellular responses, including cellular proliferation, differentiation and apoptosis (48). The activation of p38 MAPK leads to neuronal apoptosis, blood-brain barrier disruption and inflammatory responses, serving as critical contributors to secondary injury following stroke (49, 50). Specific p38 MAPK inhibitors may reduce ischemic brain injury and improve neurological recovery in animal models (51). Blocking p38 MAPK can attenuate the activation of microglia following stroke, thereby reducing the release of inflammatory cytokines (52). This is crucial in preventing the exacerbation of brain damage due to secondary inflammatory responses, implying the therapeutic potential of MAPK inhibitors such as AZD6244 in ischemic stroke.

There are several limitations of this study. A key methodological limitation concerns our approach to verifying the tMCAO model. While neurological function scoring was systematically applied to assess ischemic deficits, we did not employ laser Doppler flowmetry to objectively blood flow reduction during MCA occlusion. The absence of real-time hemodynamic confirmation introduces potential uncertainty regarding the consistency and completeness of vascular occlusion across subjects. Another limitation of this study is that RNA-seq was performed with only five subjects per group, which constrains the statistical power. To mitigate this issue and strengthen our conclusions, future prospective validation studies will incorporate larger sample sizes. In addition, our current findings are exclusively derived from *in vitro* cellular models examining FKBP5’s mechanistic role in ischemic stroke, necessitating further validation through the development of FKBP5-knockdown mouse models to substantiate these conclusions. For future research, we will increase biological replicates and incorporate prospective power analysis to optimize sample size determination.

## Conclusion

FKBP5 and CCL5 modulate p38 MAPK signaling and NET formation, contributing to post-stroke neuroinflammation and neuronal apoptosis. Further prospective research and *in vivo* experiments using FKBP5 knockout models are needed to investigate the potential of FKBP5 as therapeutic targets for ischemic stroke treatment.

## Data Availability

The original contributions presented in the study are included in the article/**Supplementary Material**. Further inquiries can be directed to the corresponding authors.
