# Correction: FKBP5-CCL5 interaction promotes neuroinflammation and neuronal apoptosis in ischemic stroke by regulating the MAPK pathway and enhancing NET formation

**DOI:** 10.3389/fimmu.2025.1744810

**Published:** 2025-12-19

**Authors:** Zhongchen Li, Tengkun Yin, Hongyang Guo, Zhenxing Liu, Peijian Wang, Chao Liu, Qingbo Wang, Meng Zhang, Yilei Xiao, Jiyue Wang, Jiheng Hao, Liyong Zhang

**Affiliations:** 1 Department of Neurosurgery, Liaocheng People’s Hospital, Liaocheng, Shandong, China; 2 Department of Neurosurgery, Qilu Hospital and Institute of Brain and Brain-Inspired Science, Cheeloo College of Medicine, Shandong University, Shandong, China

**Keywords:** ischemic stroke, FKBP5, CCL5, NET, MAPK

Affiliation “Department of Neurosurgery, Qilu Hospital and Institute of Brain and Brain-Inspired Science, Cheeloo College of Medicine, Shandong University, Shandong, China” was omitted for author “Tengkun Yin”. This affiliation has now been added for author “Tengkun Yin”.

Also, there was a mistake in **Figure 7B** as published. Upon reviewing the published version, we noticed that **Figure 7B** was inadvertently placed error, which was the same as **Figure 7A**. The corrected **Figure 7B** appears below.

**Figure 7:**
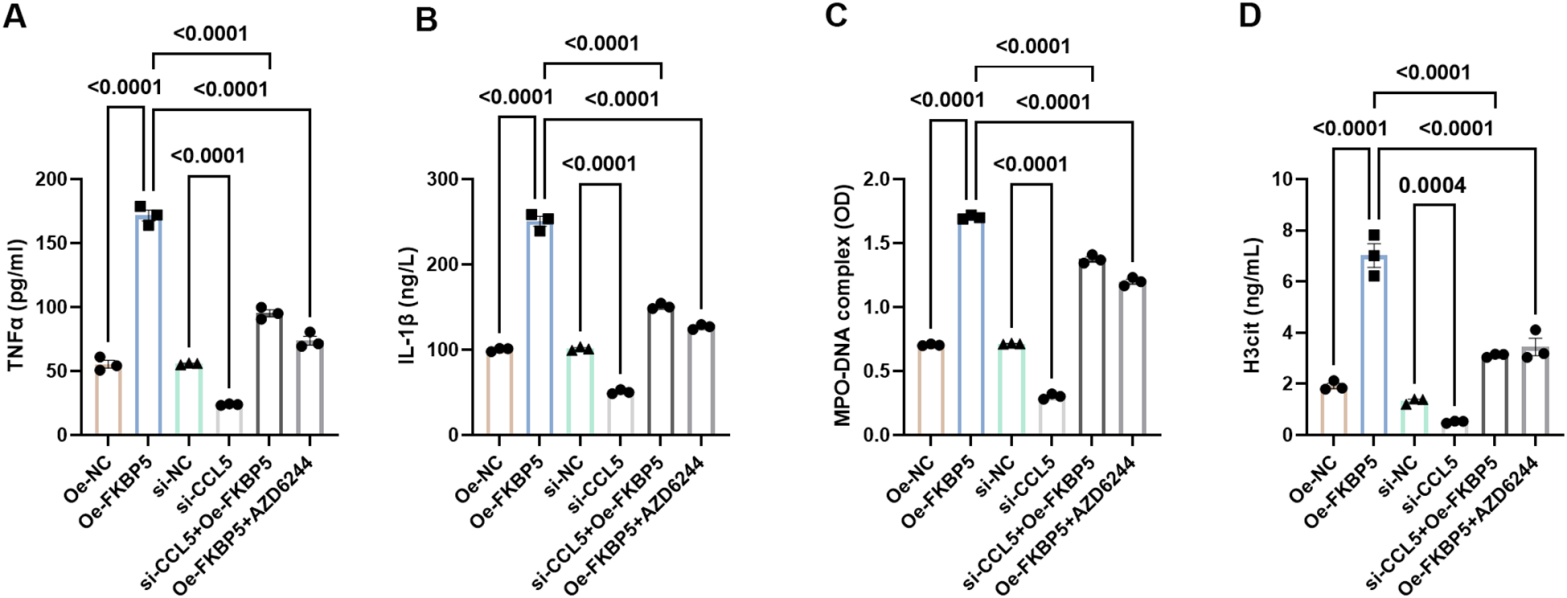
Influence of FKBP5 on BV2 cell polarization and neutrophil NETs formation via the p38 MAPK signaling pathway. Levels of pro-inflammatory cytokines, TNFα **(A)** and IL-1β **(B)**, were quantified using ELISA in different groups. Levels of NET markers, MPO-DNA **(C)** and H3cit **(D)**, were quantified using ELISA in different groups. AZD6244 is an inhibitor of the MAPK pathway. n = 3.

The original version of this article has been updated.

