# Supplementary figures and images for: FKBP5-CCL5 interaction promotes neuroinflammation and neuronal apoptosis in ischemic stroke by regulating the MAPK pathway and enhancing NET formation
Source: Front Immunol. 2025 Sep 30;16:1609989. doi: 10.3389/fimmu.2025.1609989 (PMC12518073; doi:10.3389/fimmu.2025.1609989)

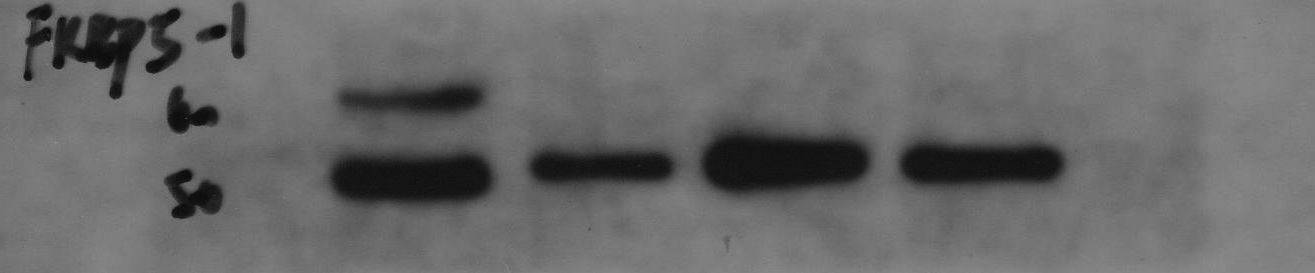

Supplement: Supplementary file 1 [file DataSheet1.zip › WB Gels and Blots images/Figure4/4B/FKBP5-1.jpg]

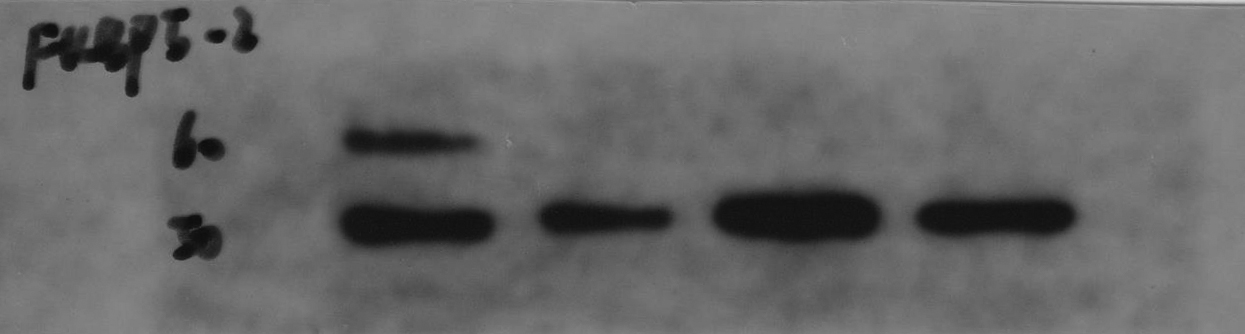

Supplement: Supplementary file 1 [file DataSheet1.zip › WB Gels and Blots images/Figure4/4B/FKBP5-2.jpg]

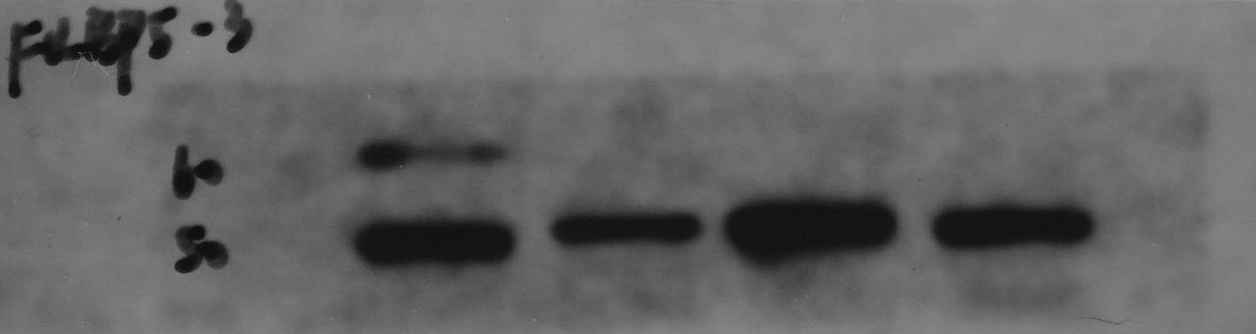

Supplement: Supplementary file 1 [file DataSheet1.zip › WB Gels and Blots images/Figure4/4B/FKBP5-3.jpg]

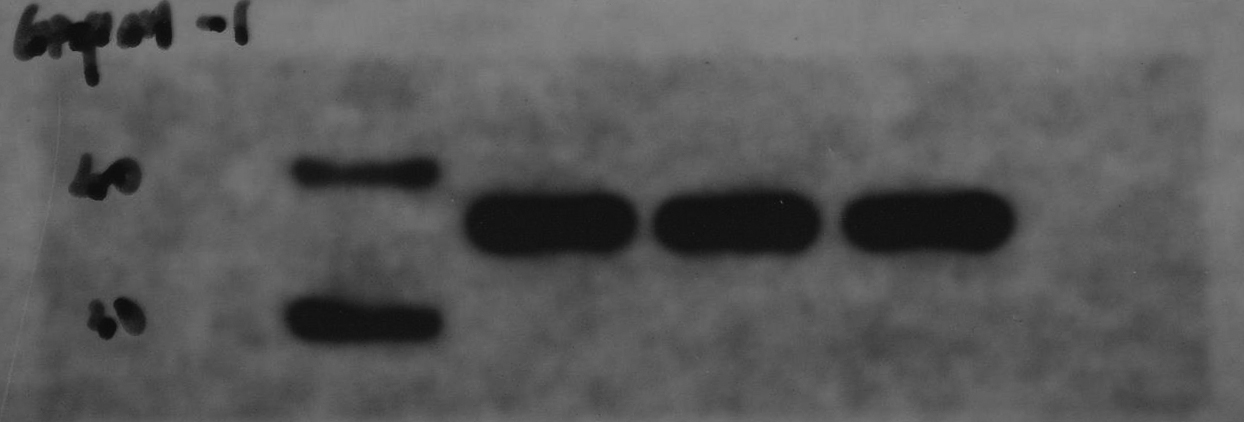

Supplement: Supplementary file 1 [file DataSheet1.zip › WB Gels and Blots images/Figure4/4B/GAPDH-1.jpg]

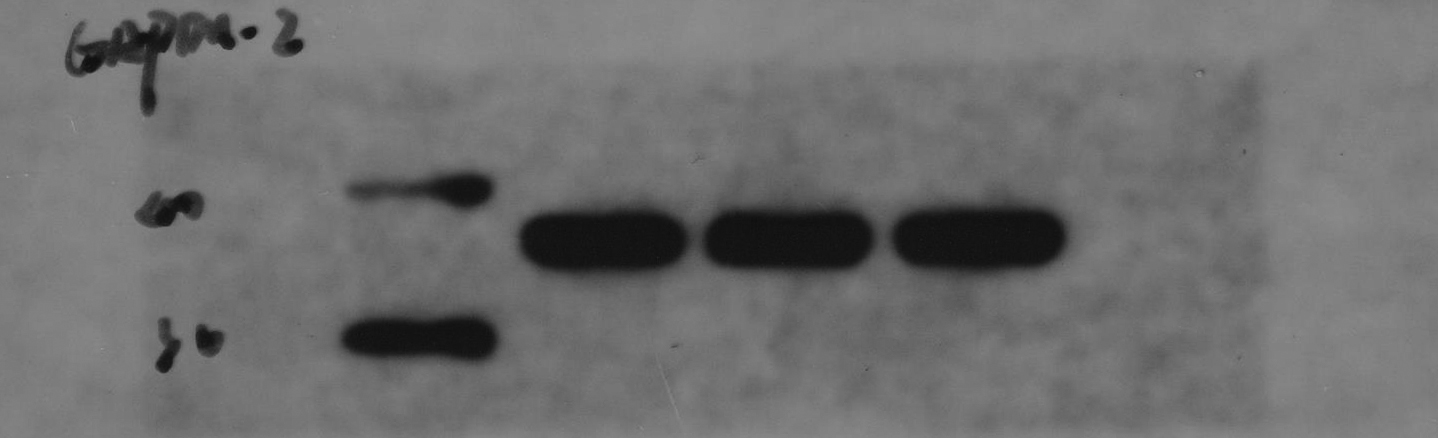

Supplement: Supplementary file 1 [file DataSheet1.zip › WB Gels and Blots images/Figure4/4B/GAPDH-2.jpg]

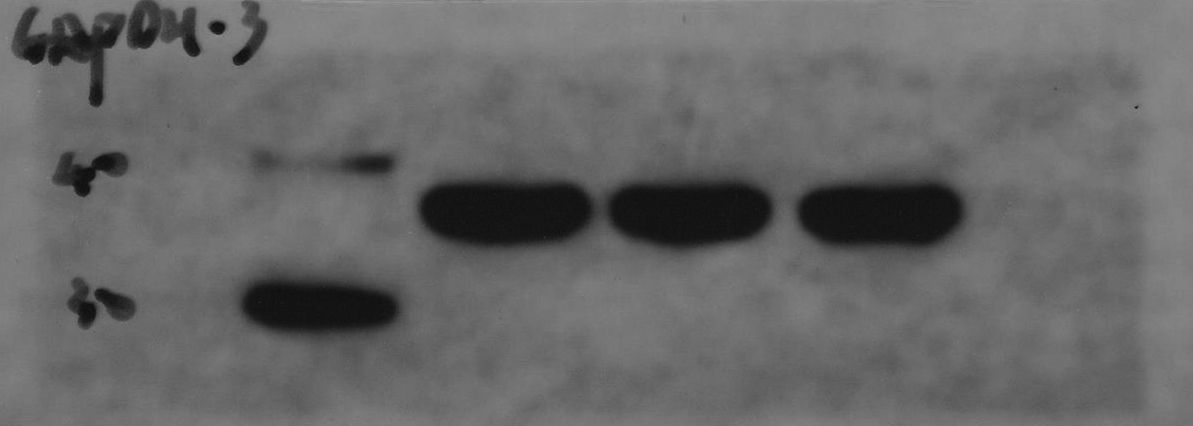

Supplement: Supplementary file 1 [file DataSheet1.zip › WB Gels and Blots images/Figure4/4B/GAPDH-3.jpg]

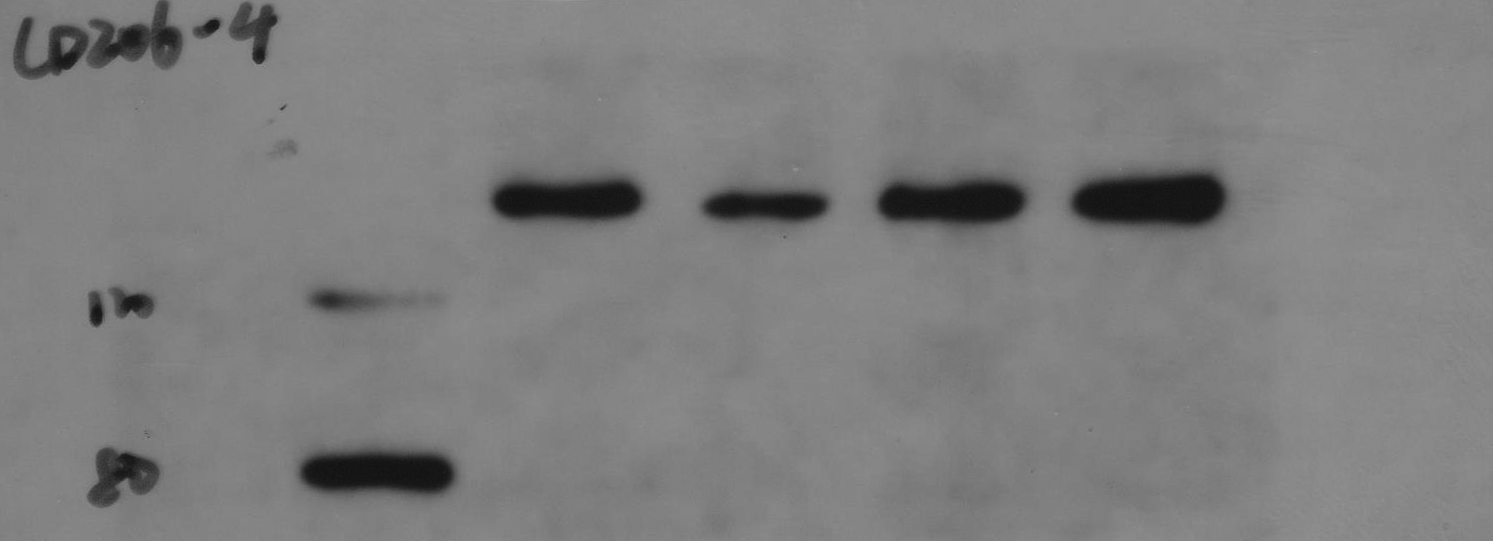

Supplement: Supplementary file 1 [file DataSheet1.zip › WB Gels and Blots images/Figure4/4H/CD206-1.jpg]

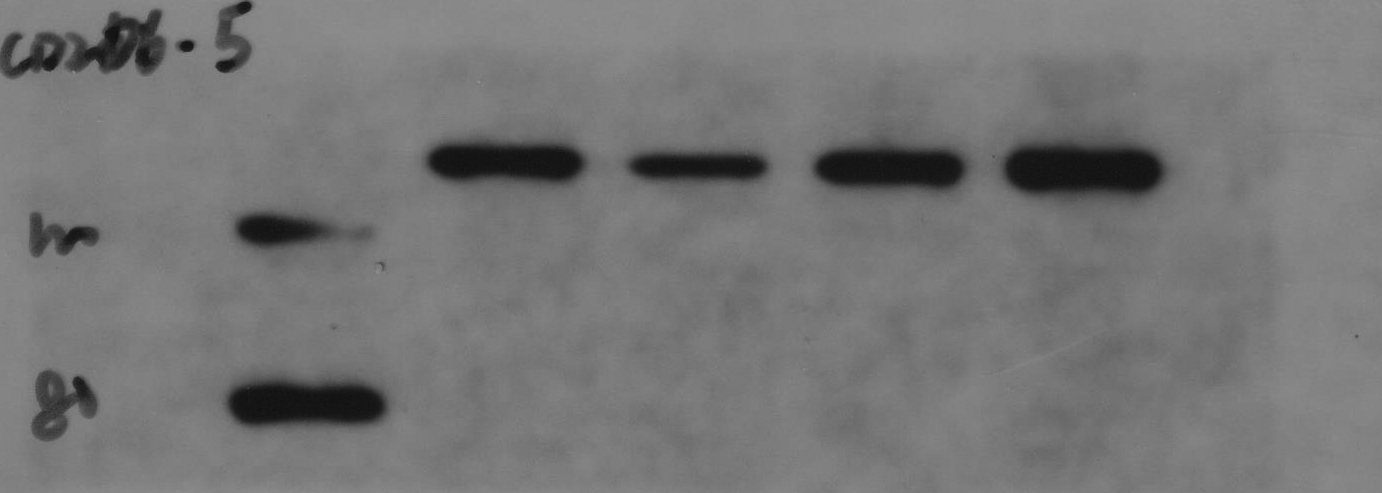

Supplement: Supplementary file 1 [file DataSheet1.zip › WB Gels and Blots images/Figure4/4H/CD206-2.jpg]

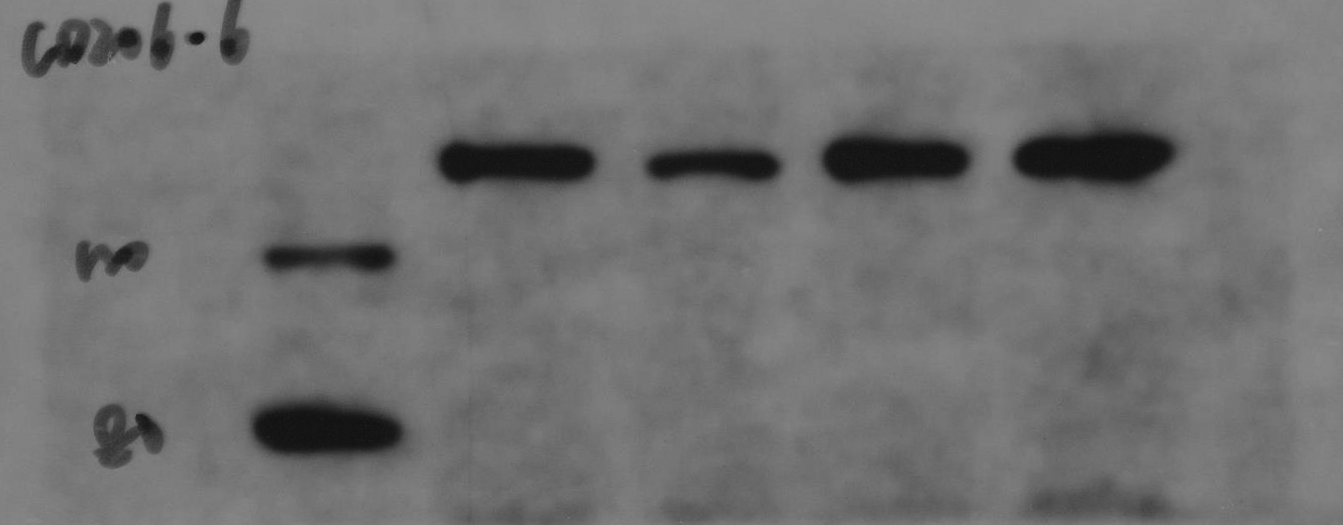

Supplement: Supplementary file 1 [file DataSheet1.zip › WB Gels and Blots images/Figure4/4H/CD206-3.jpg]

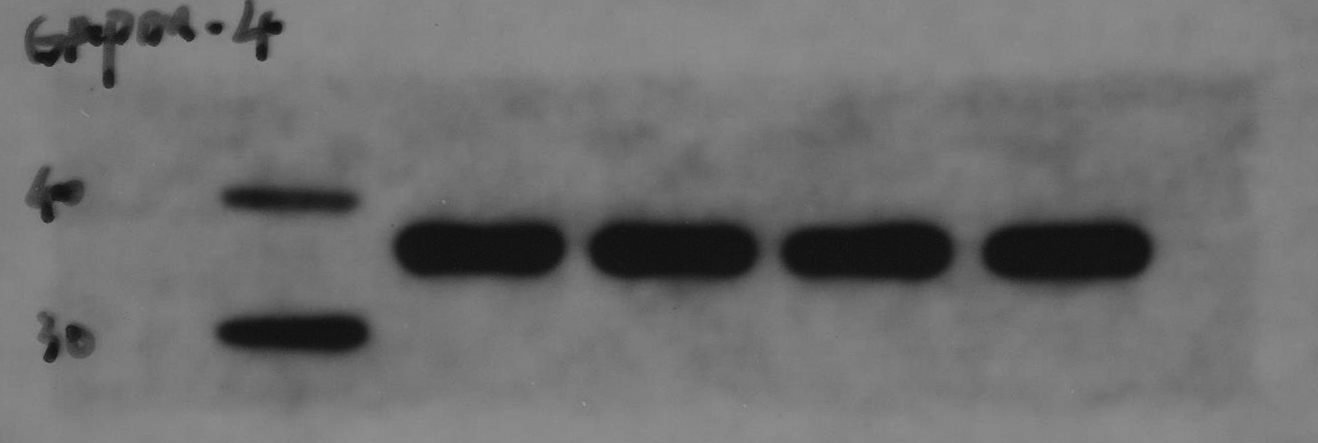

Supplement: Supplementary file 1 [file DataSheet1.zip › WB Gels and Blots images/Figure4/4H/GAPDH-1.jpg]

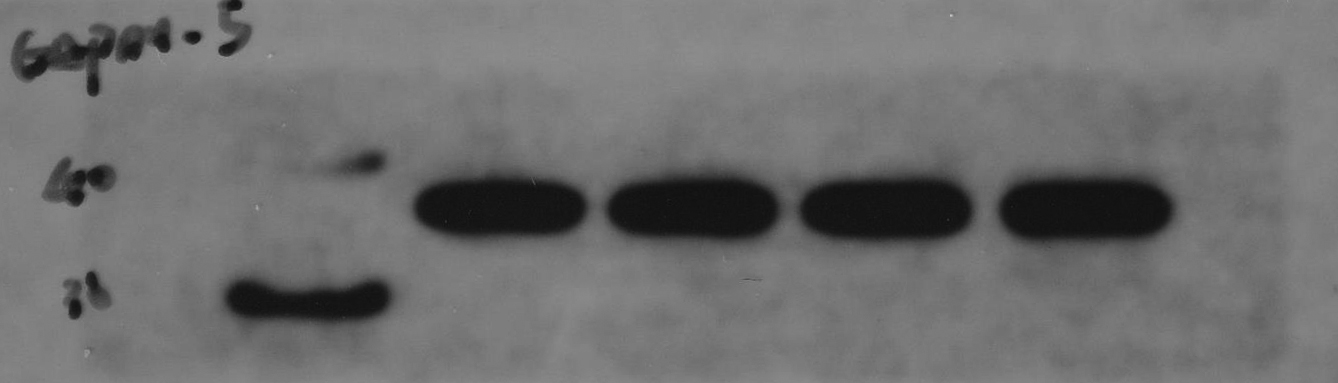

Supplement: Supplementary file 1 [file DataSheet1.zip › WB Gels and Blots images/Figure4/4H/GAPDH-2.jpg]

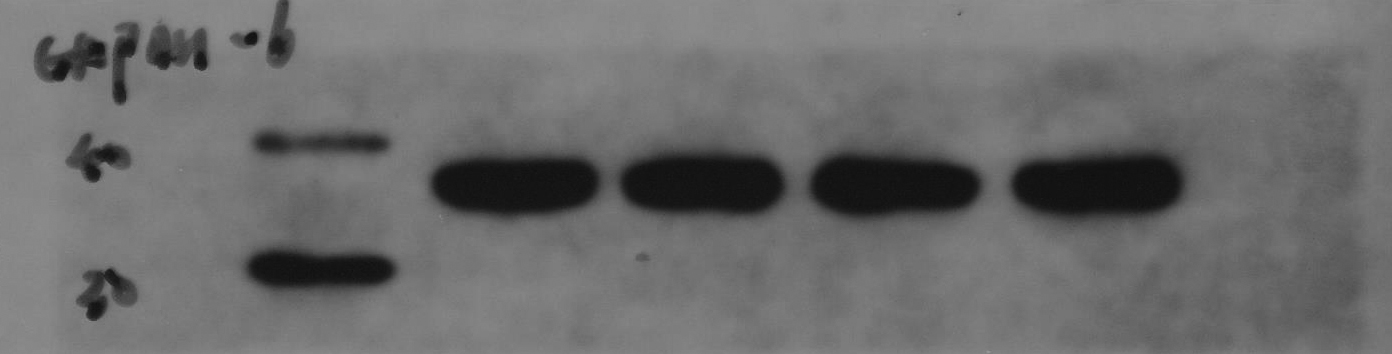

Supplement: Supplementary file 1 [file DataSheet1.zip › WB Gels and Blots images/Figure4/4H/GAPDH-3.jpg]

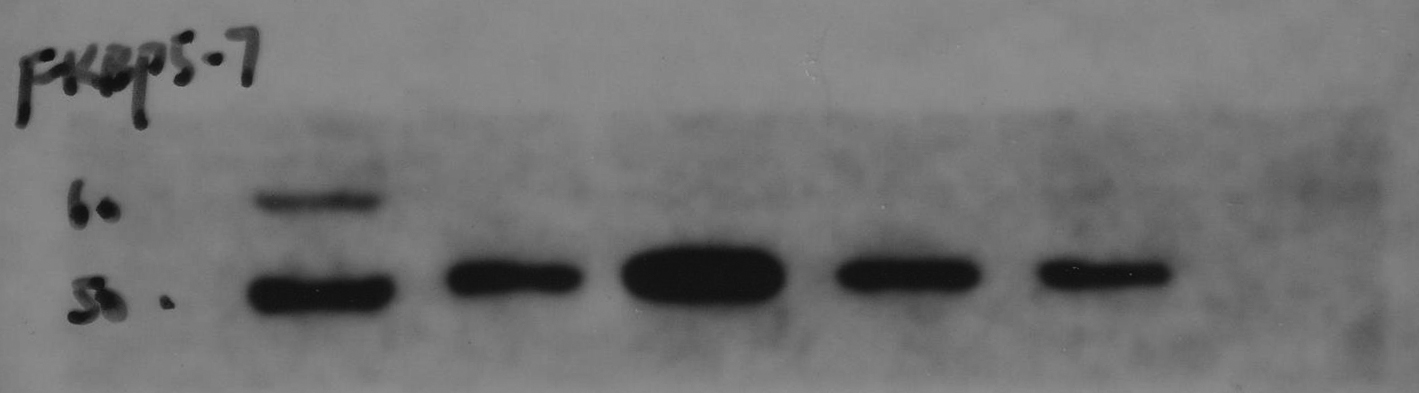

Supplement: Supplementary file 1 [file DataSheet1.zip › WB Gels and Blots images/Figure5/FKBP5-1.jpg]

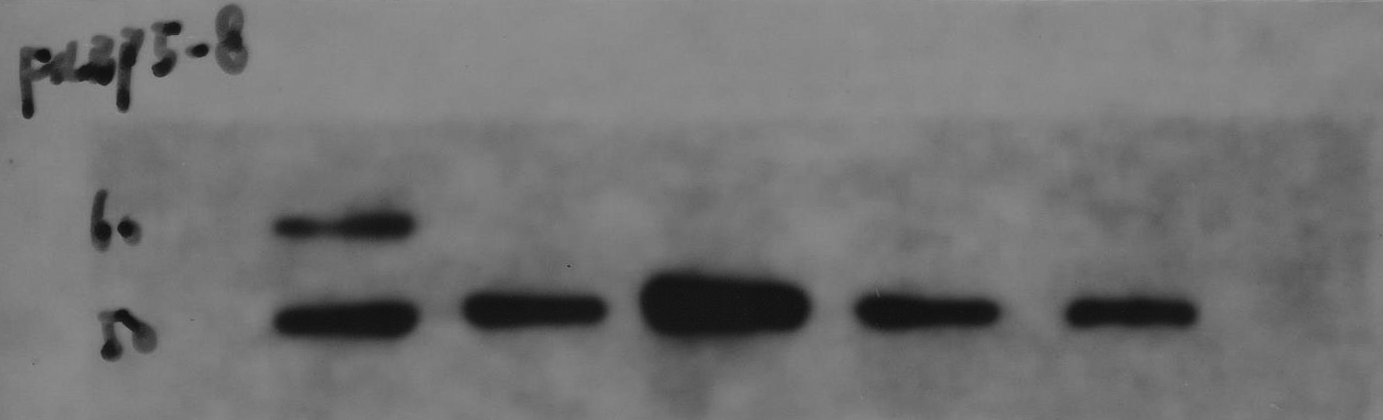

Supplement: Supplementary file 1 [file DataSheet1.zip › WB Gels and Blots images/Figure5/FKBP5-2.jpg]

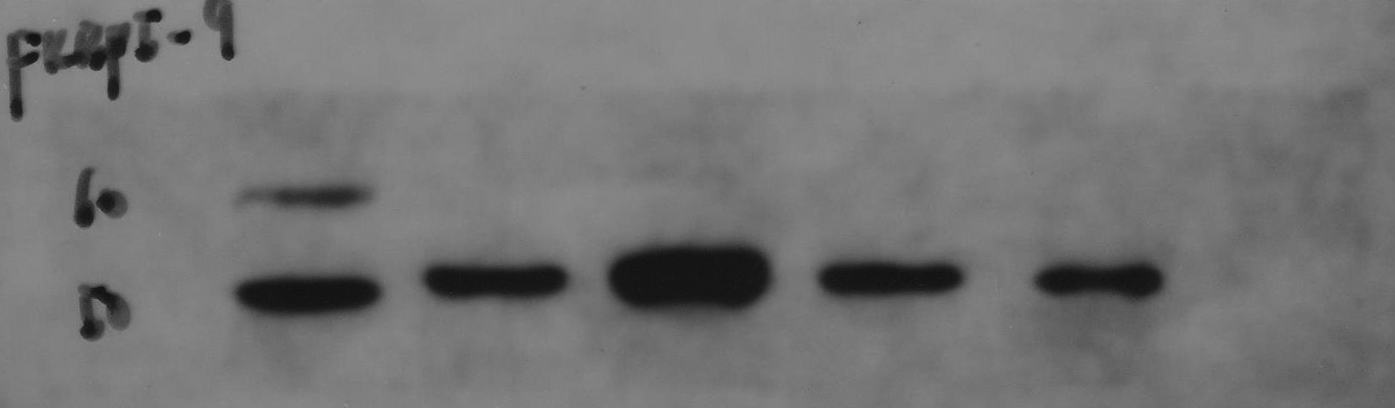

Supplement: Supplementary file 1 [file DataSheet1.zip › WB Gels and Blots images/Figure5/FKBP5-3.jpg]

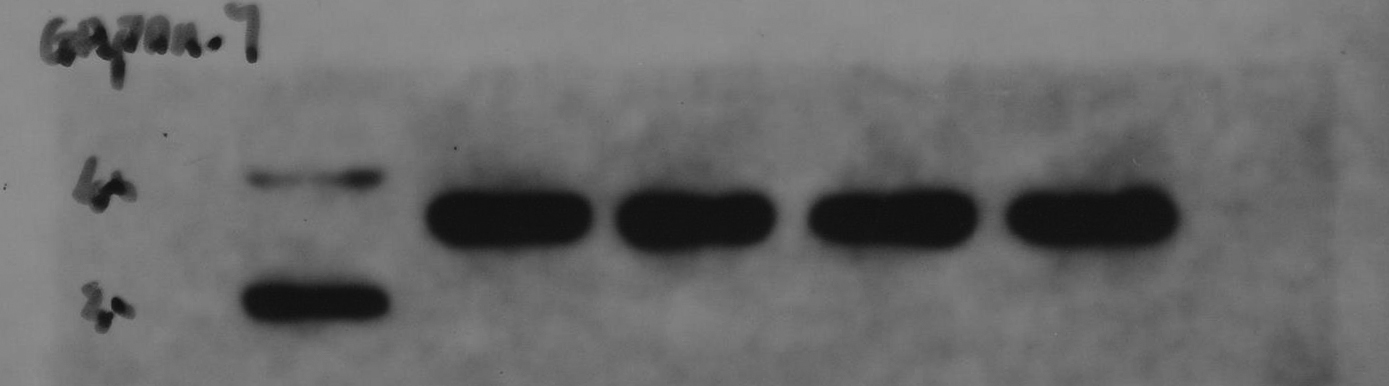

Supplement: Supplementary file 1 [file DataSheet1.zip › WB Gels and Blots images/Figure5/GAPDH-1.jpg]

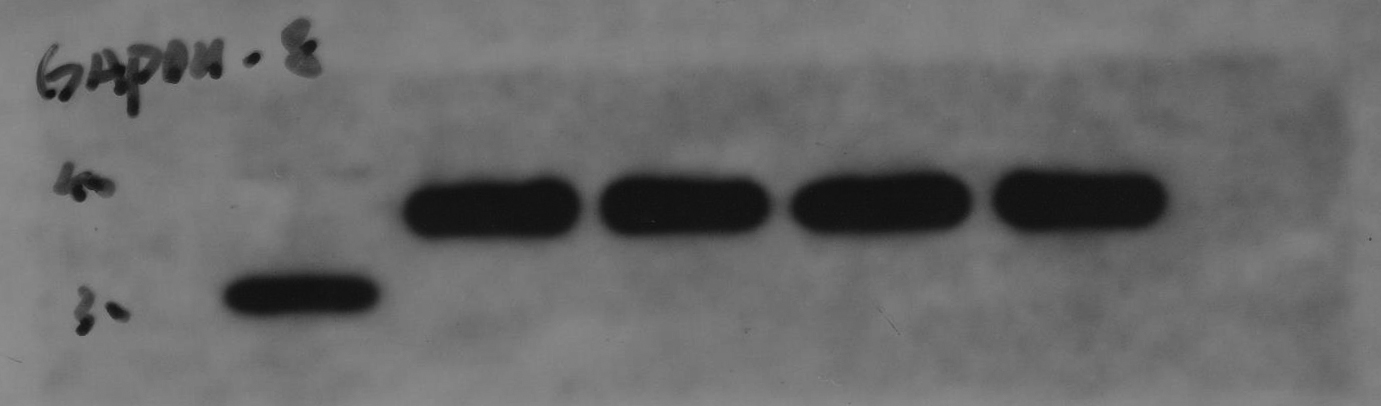

Supplement: Supplementary file 1 [file DataSheet1.zip › WB Gels and Blots images/Figure5/GAPDH-2.jpg]

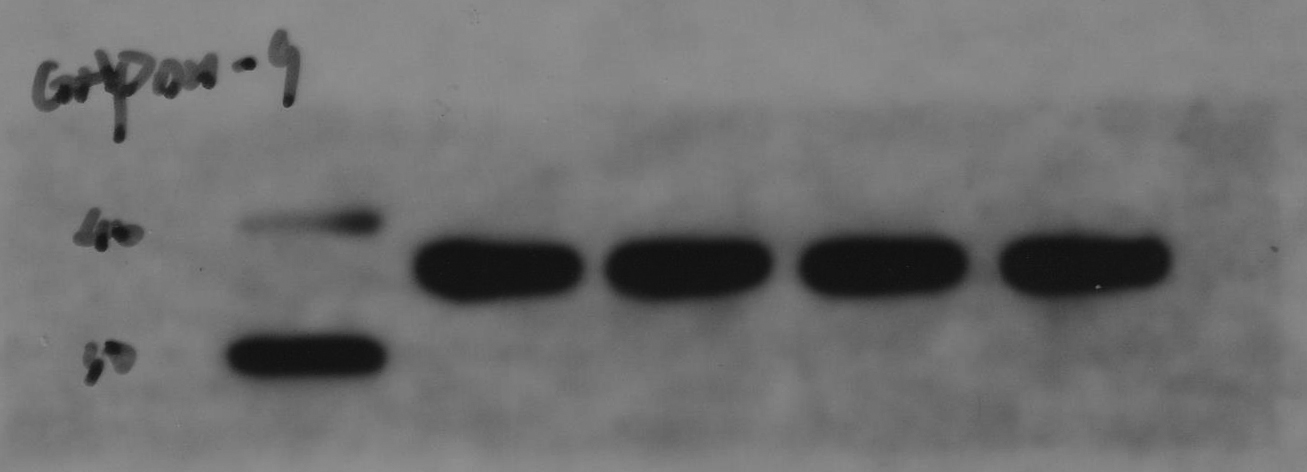

Supplement: Supplementary file 1 [file DataSheet1.zip › WB Gels and Blots images/Figure5/GAPDH-3.jpg]

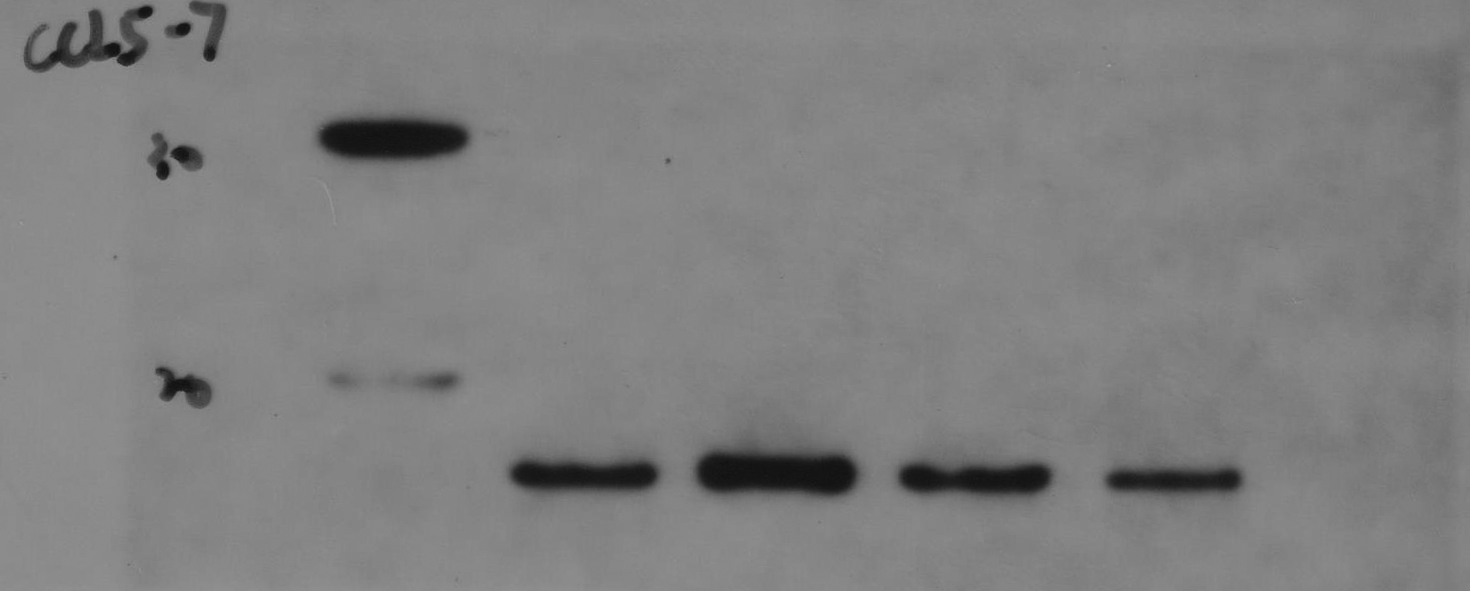

Supplement: Supplementary file 1 [file DataSheet1.zip › WB Gels and Blots images/Figure5/ccl5-1.jpg]

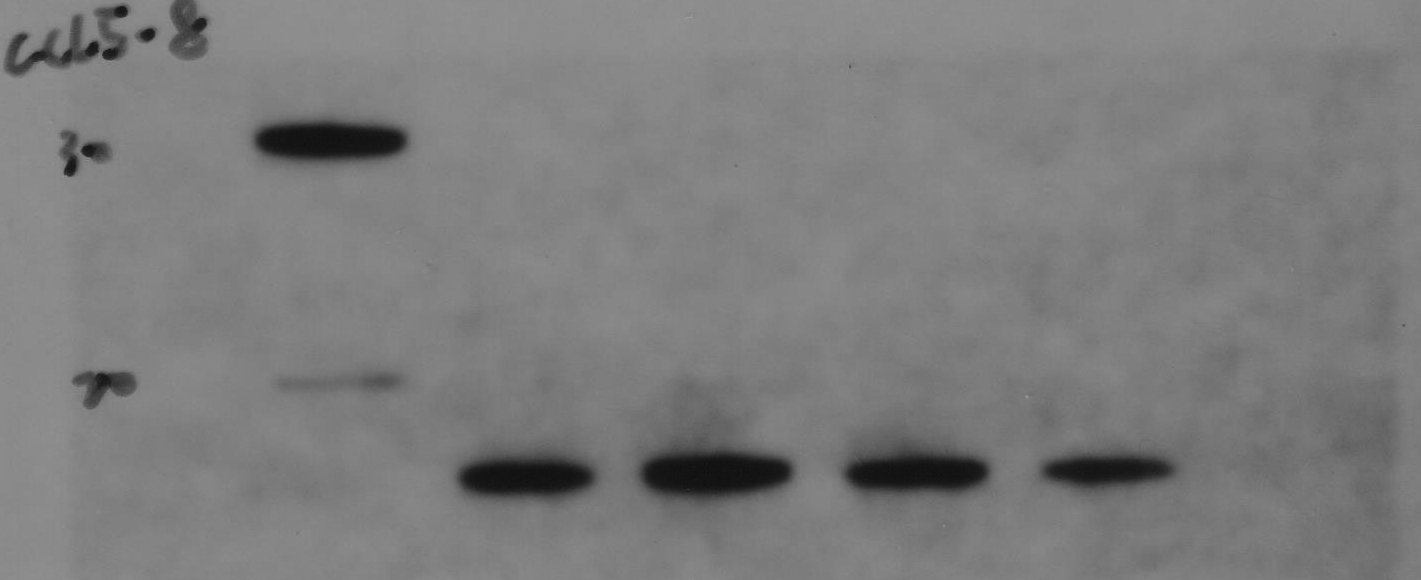

Supplement: Supplementary file 1 [file DataSheet1.zip › WB Gels and Blots images/Figure5/ccl5-2.jpg]

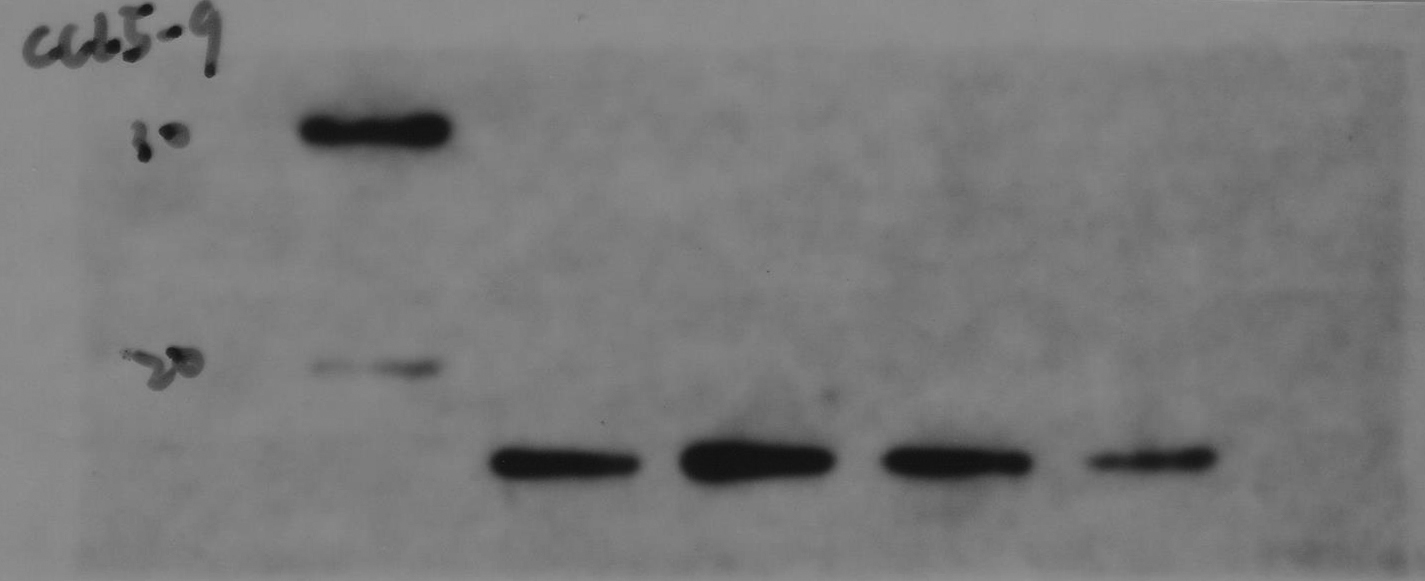

Supplement: Supplementary file 1 [file DataSheet1.zip › WB Gels and Blots images/Figure5/ccl5-3.jpg]

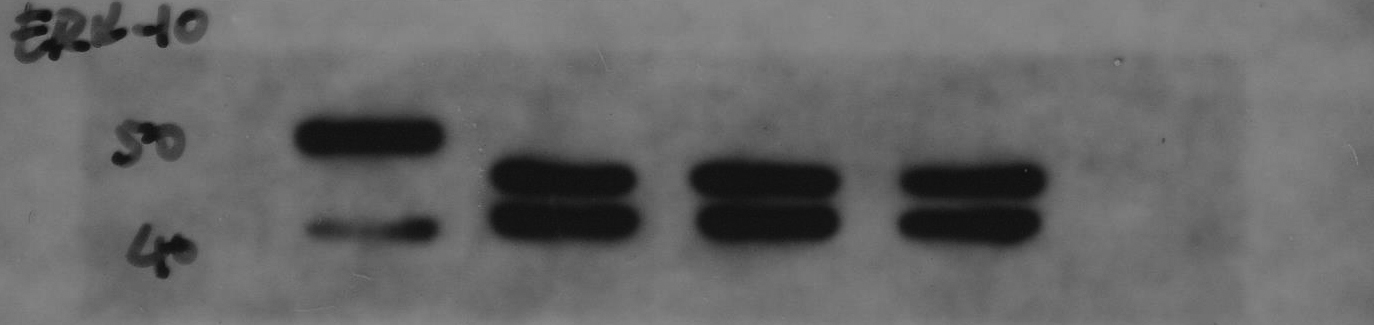

Supplement: Supplementary file 1 [file DataSheet1.zip › WB Gels and Blots images/Figure6/ERK-1.jpg]

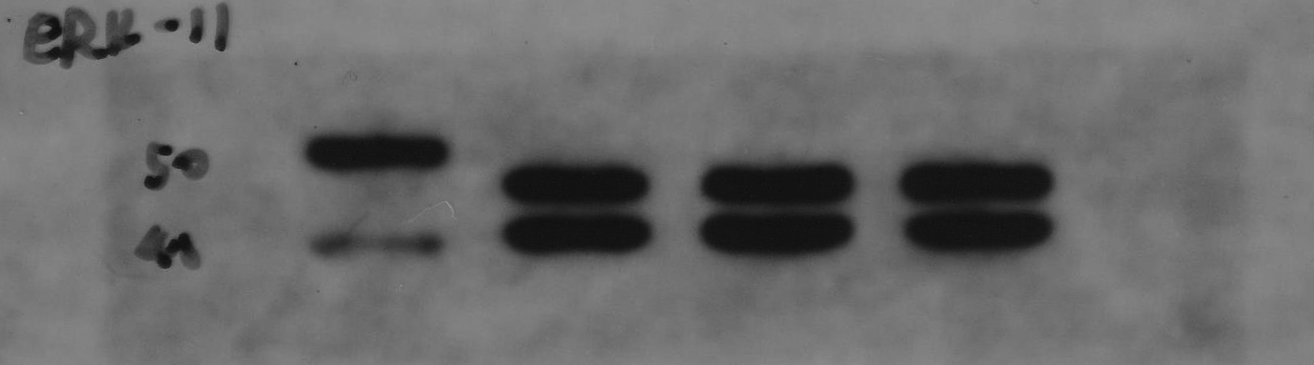

Supplement: Supplementary file 1 [file DataSheet1.zip › WB Gels and Blots images/Figure6/ERK-2.jpg]

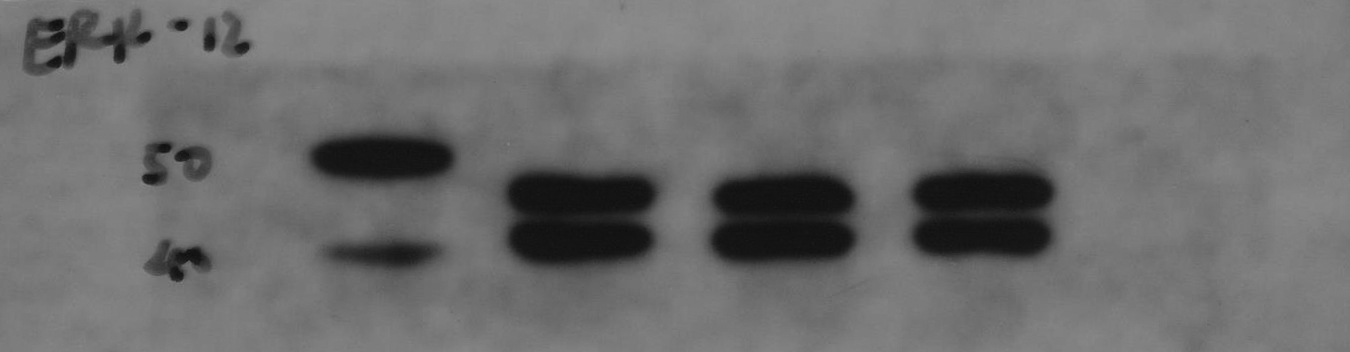

Supplement: Supplementary file 1 [file DataSheet1.zip › WB Gels and Blots images/Figure6/ERK-3.jpg]

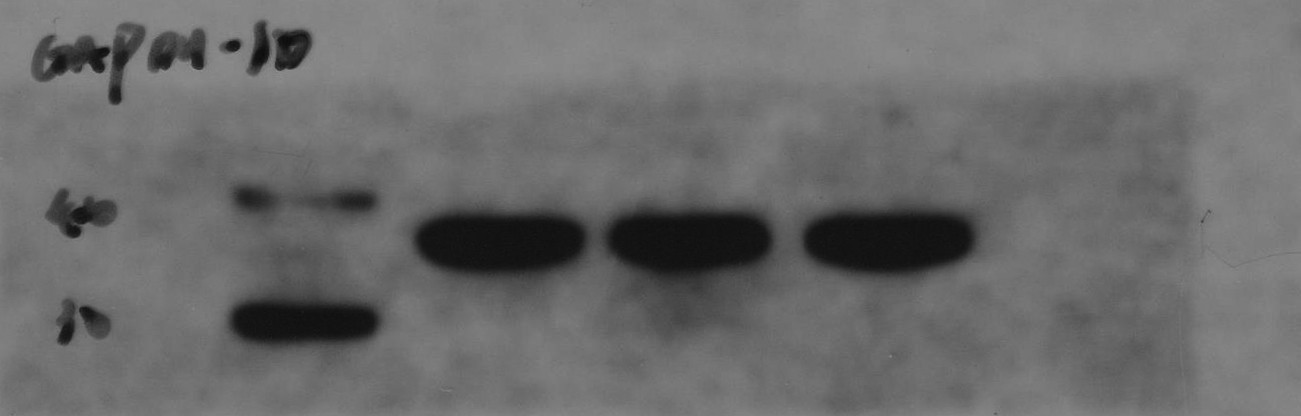

Supplement: Supplementary file 1 [file DataSheet1.zip › WB Gels and Blots images/Figure6/GAPDH-1.jpg]

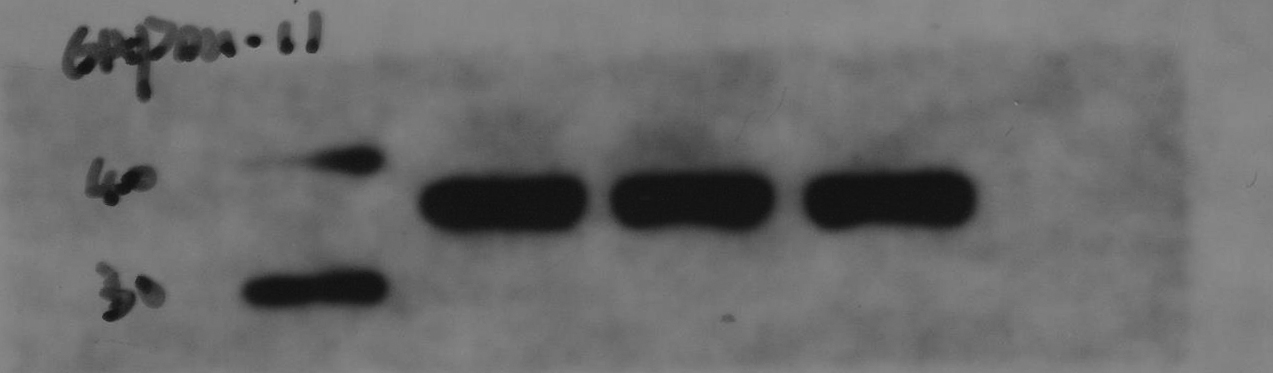

Supplement: Supplementary file 1 [file DataSheet1.zip › WB Gels and Blots images/Figure6/GAPDH-2.jpg]

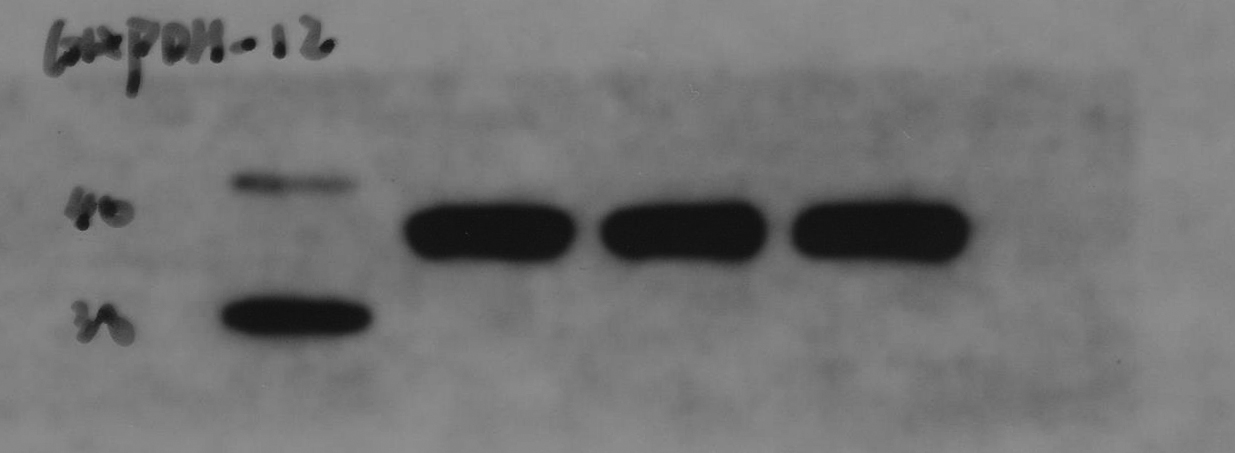

Supplement: Supplementary file 1 [file DataSheet1.zip › WB Gels and Blots images/Figure6/GAPDH-3.jpg]

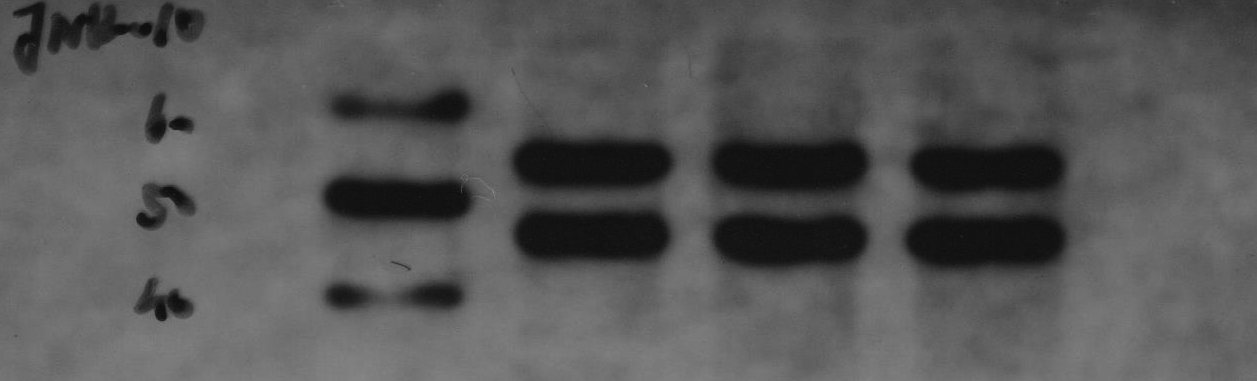

Supplement: Supplementary file 1 [file DataSheet1.zip › WB Gels and Blots images/Figure6/JNK-1.jpg]

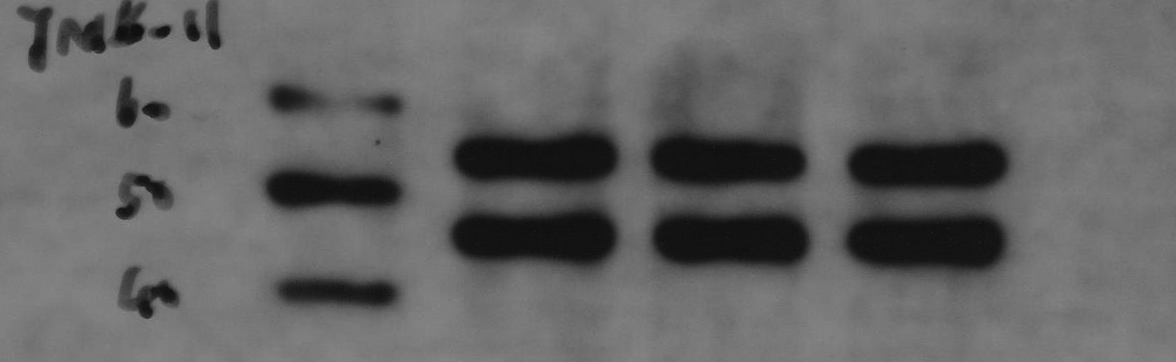

Supplement: Supplementary file 1 [file DataSheet1.zip › WB Gels and Blots images/Figure6/JNK-2.jpg]

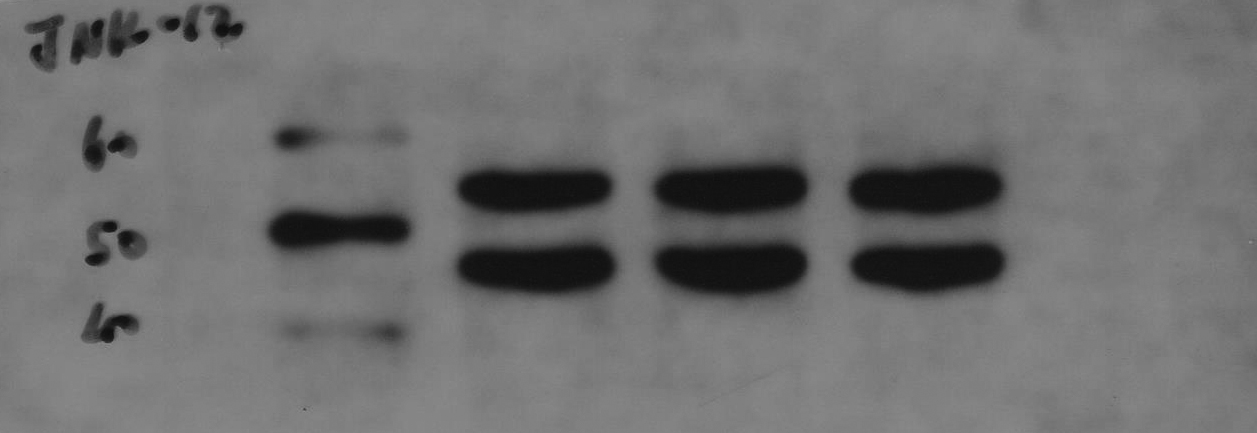

Supplement: Supplementary file 1 [file DataSheet1.zip › WB Gels and Blots images/Figure6/JNK-3.jpg]

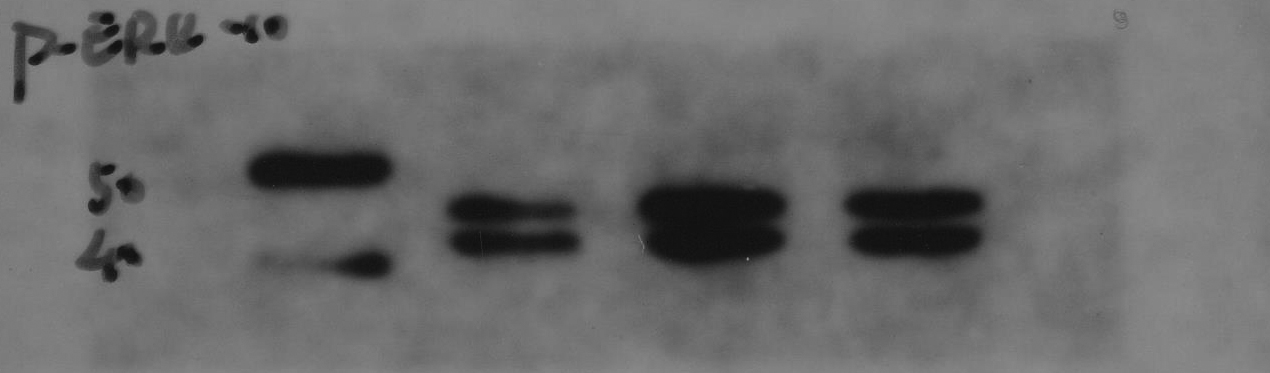

Supplement: Supplementary file 1 [file DataSheet1.zip › WB Gels and Blots images/Figure6/P-ERK-1.jpg]

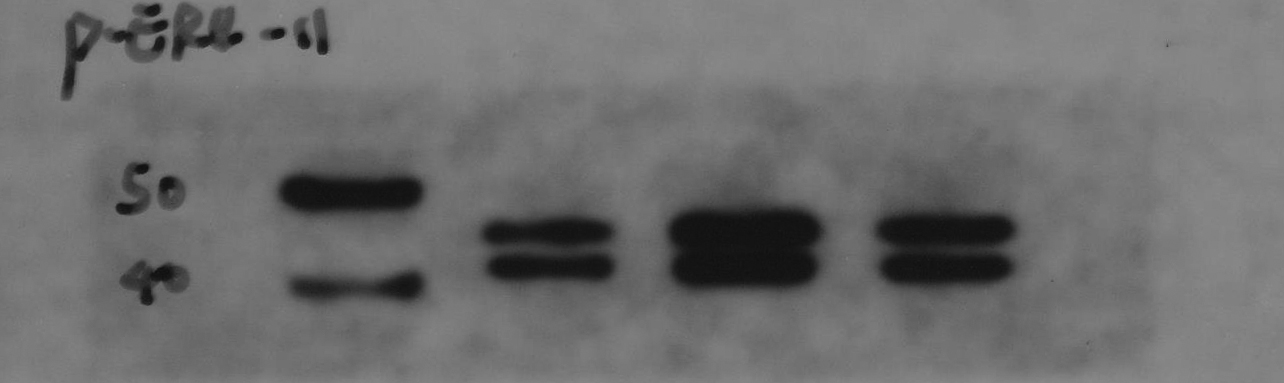

Supplement: Supplementary file 1 [file DataSheet1.zip › WB Gels and Blots images/Figure6/P-ERK-2.jpg]

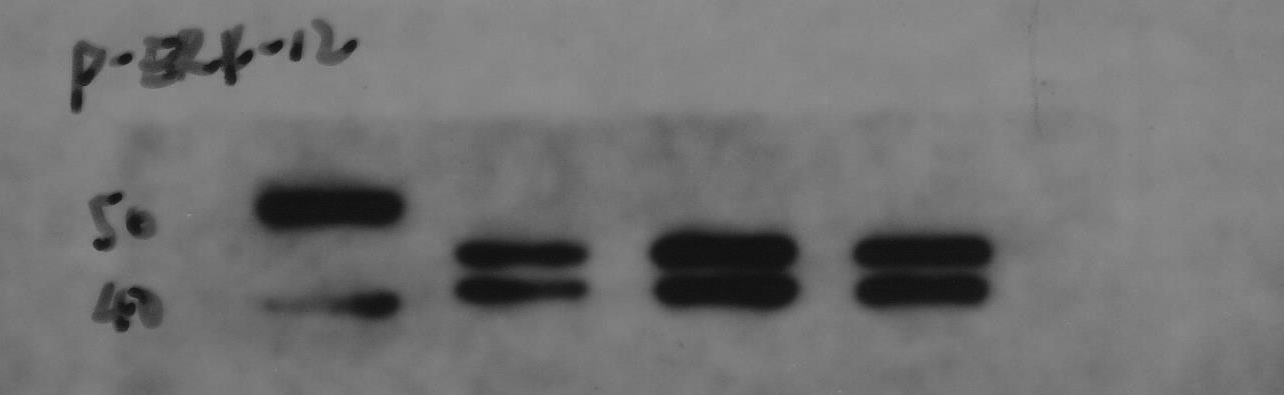

Supplement: Supplementary file 1 [file DataSheet1.zip › WB Gels and Blots images/Figure6/P-ERK-3.jpg]

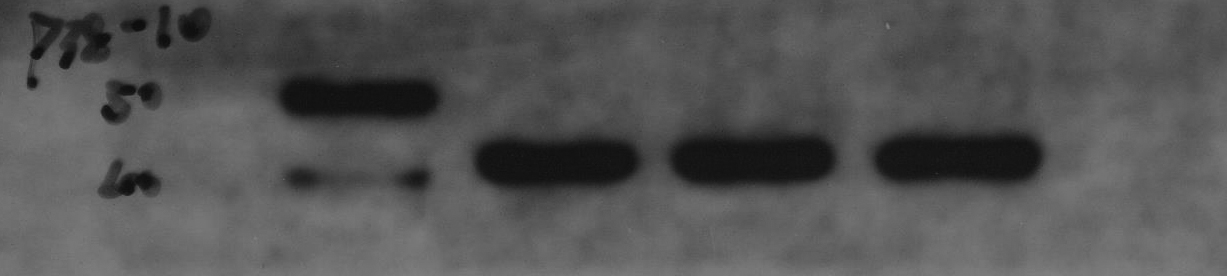

Supplement: Supplementary file 1 [file DataSheet1.zip › WB Gels and Blots images/Figure6/P38-1.jpg]

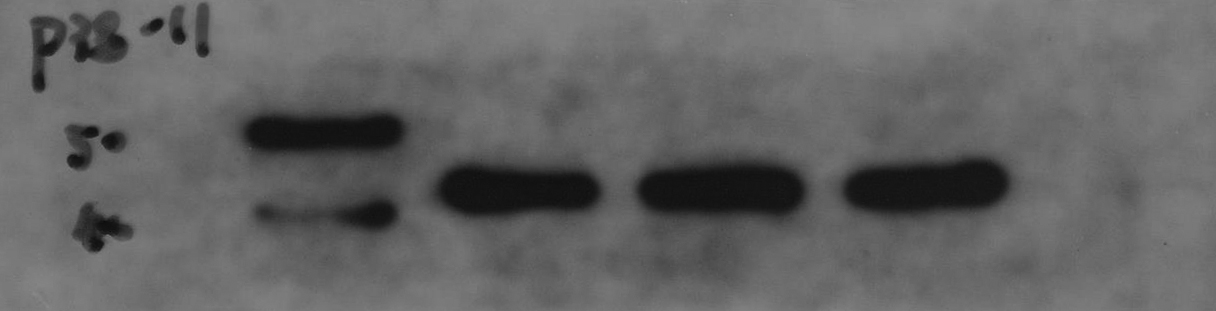

Supplement: Supplementary file 1 [file DataSheet1.zip › WB Gels and Blots images/Figure6/P38-2.jpg]

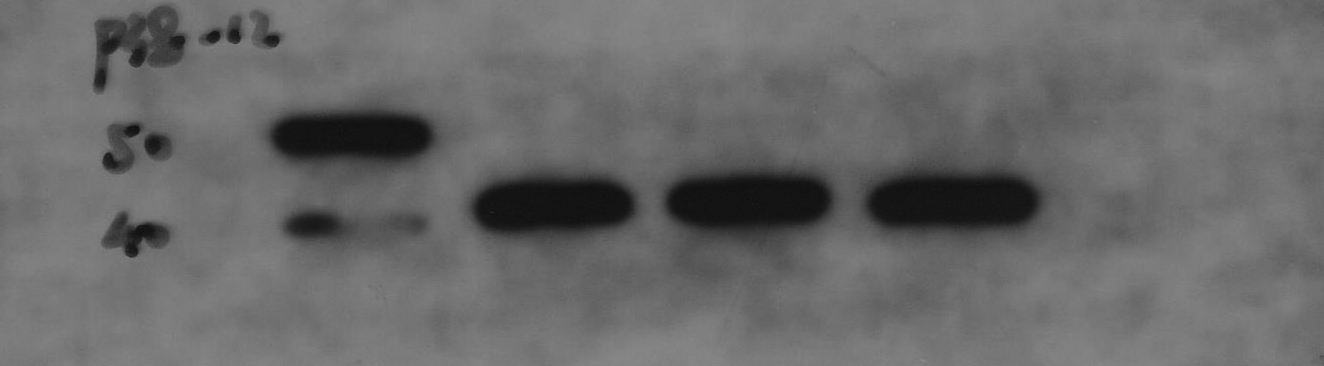

Supplement: Supplementary file 1 [file DataSheet1.zip › WB Gels and Blots images/Figure6/P38-3.jpg]

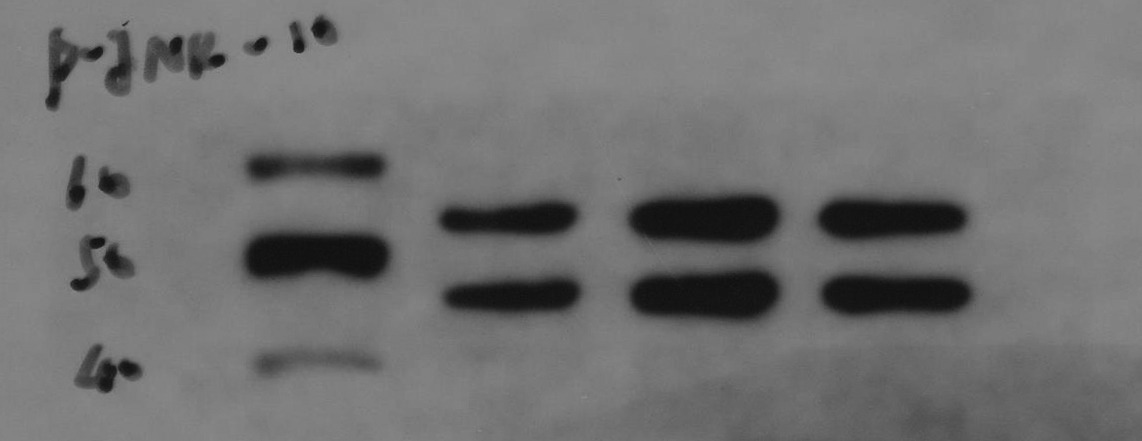

Supplement: Supplementary file 1 [file DataSheet1.zip › WB Gels and Blots images/Figure6/p-JNK-1.jpg]

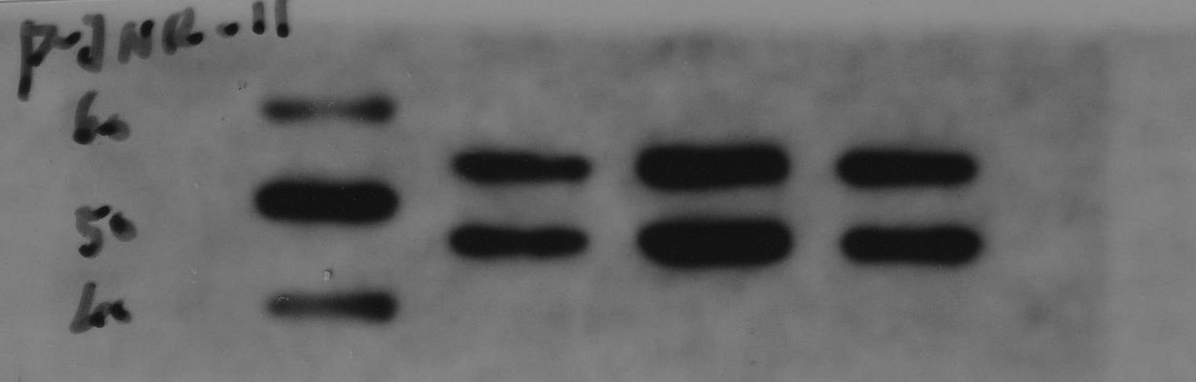

Supplement: Supplementary file 1 [file DataSheet1.zip › WB Gels and Blots images/Figure6/p-JNK-2.jpg]

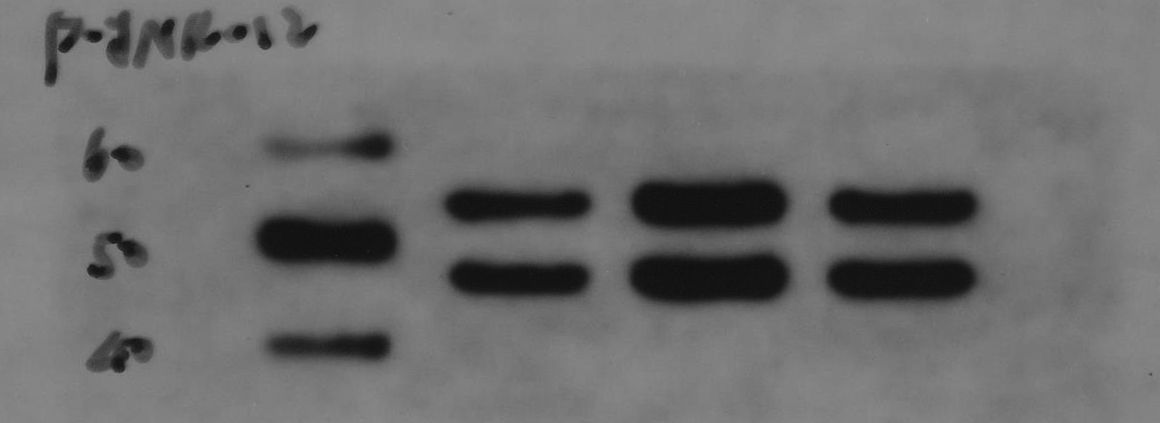

Supplement: Supplementary file 1 [file DataSheet1.zip › WB Gels and Blots images/Figure6/p-JNK-3.jpg]

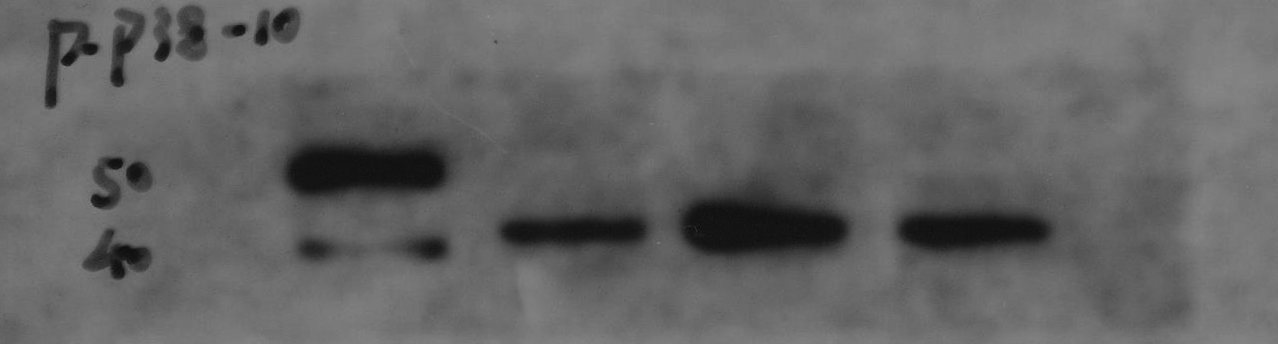

Supplement: Supplementary file 1 [file DataSheet1.zip › WB Gels and Blots images/Figure6/p-P38-1.jpg]

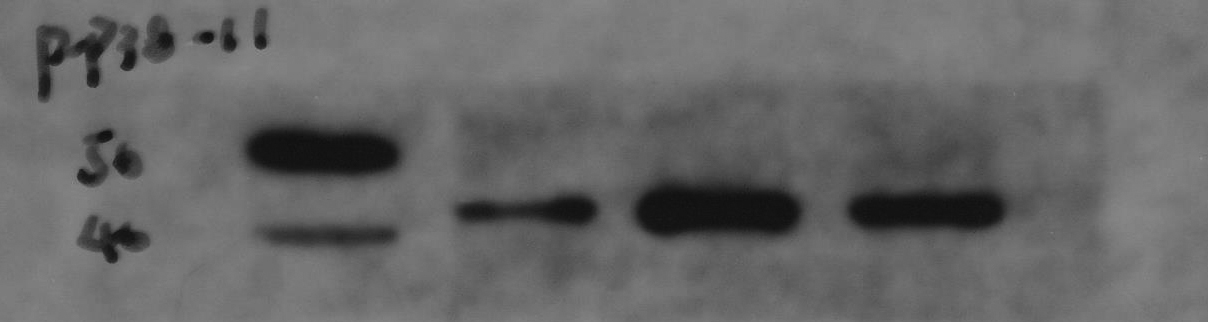

Supplement: Supplementary file 1 [file DataSheet1.zip › WB Gels and Blots images/Figure6/p-P38-2.jpg]

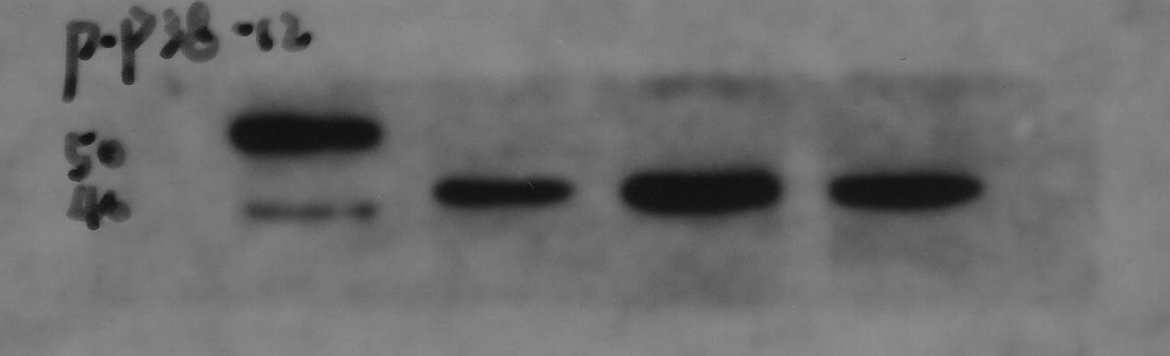

Supplement: Supplementary file 1 [file DataSheet1.zip › WB Gels and Blots images/Figure6/p-P38-3.jpg]
